# Essential oils and their active components applied as: free, encapsulated and in hurdle technology to fight microbial contaminations. A review

**DOI:** 10.1016/j.heliyon.2022.e12472

**Published:** 2022-12-20

**Authors:** Jina Yammine, Nour-Eddine Chihib, Adem Gharsallaoui, Emilie Dumas, Ali Ismail, Layal Karam

**Affiliations:** a Univ Lille, CNRS, INRAE, Centrale Lille, UMR 8207 – UMET – Unité Matériaux et Transformations, Lille, France; b Univ Lyon, Université Claude Bernard Lyon 1, CNRS, LAGEPP UMR 5007, Villeurbanne, France; c Plateforme de Recherches et d’Analyses en Sciences de l’Environnement (PRASE), Ecole Doctorale des Sciences et Technologies, Université Libanaise, Hadath, Lebanon; d Human Nutrition Department, College of Health Sciences, QU Health, Qatar University, Doha, Qatar

**Keywords:** Essential oils, Antimicrobial activity, Encapsulation, Hurdle technology, Disinfection

## Abstract

Microbial contaminations are responsible for many chronic, healthcare, persistent microbial infections and illnesses in the food sector, therefore their control is an important public health challenge. Over the past few years, essential oils (EOs) have emerged as interesting alternatives to synthetic antimicrobials as they are biodegradable, extracted from natural sources and potent antimicrobials. Through their multiple mechanisms of actions and target sites, no microbial resistance has been developed against them till present. Although extensive documentation has been reported on the antimicrobial activity of EOs, comparisons between the use of whole EOs or their active components alone for an antimicrobial treatment are less abundant. It is also essential to have a good knowledge about EOs to be used as alternatives to the conventional antimicrobial products such as chemical disinfectants. Moreover, it is important to focus not only on planktonic vegetative microorganisms, but to study also the effect on more resistant forms like spores and biofilms. The present article reviews the current knowledge on the mechanisms of antimicrobial activities of EOs and their active components on microorganisms in different forms. Additionally, in this review, the ultimate advantages of encapsulating EOs or combining them with other hurdles for enhanced antimicrobial treatments are discussed.

## 1 Introduction

The persistence of different types of microbial contaminations including vegetative, viable but nonculturable, spores, planktonic and sessile cells, remains a serious public health concern in food, industrial, biomedical and environmental fields despite the different prevention and control measures. These contaminations and their subsequent consequences result in an increase of occurrence of food-borne diseases, outbreaks, reduced shelf-life of food products, costly socio-economical losses, along with healthcare-associated infections. The centers for disease control (CDC) estimates that annually, around 48 million Americans suffer from foodborne illnesses resulting in 3000 deaths and 128,000 hospitalizations (Scharff, 2012). The European Food Safety Authority (EFSA) and the European Center for Disease Prevention and Control (ECDC) reported in 2018 a total of 5079 food-borne and water-borne outbreaks in the European Union (EU) (EFSA and ECDC, 2018). Along the health losses, foodborne illnesses have created economic burdens from which, illness costs are estimated to be annually between $60.9 and $90.2 billion in the United States (Scharff, 2018).

Several synthetic antimicrobials and control measures have been adopted to overcome microbial contaminations. Nevertheless, due to the recent negative perception of consumers towards synthetic antimicrobials, the resistance developed by certain microorganisms towards them, their possible side toxic effects along with their low biodegradability and negative environmental impacts, increased efforts are urgently needed to find some new effective control measures to limit microbial contaminations (Campana et al., 2017; Calo et al., 2015).

In recent years, biosourced antimicrobials and particularly essential oils (EOs) and their active components have received remarkable attention and emerged as vigorous and convenient natural alternatives for synthetic antimicrobials. For many years, EOs have been used in therapies, pharmaceuticals, perfumes, cosmetics and in food industries. They are environmental-friendly, biodegradable into non-toxic products with potent antimicrobial activity against different types of microbial contaminations (Ju et al., 2019; Teixeira et al., 2022). Additionally, several EO active components have been recognized as GRAS (Generally Recognized as Safe) by the US Food and Drug Administration (FDA) and have been accepted by the European Commission as flavoring agents in food products (Gutiérrez-del-Río et al., 2018). Till present, most of literature studies demonstrated no particular antimicrobial resistance to EOs as they exhibit several modes of action against multiple target sites in bacterial cells (García-Salinas et al., 2018; Vidács et al., 2018). Few studies reported a variable sensitivity of bacterial strains to EOs and/or an emergence of an antimicrobial resistance (Becerril et al., 2012; Marotta et al., 2016). However, further exploration is still required to have a strong evidence about the potential resistance that could be developed by the different bacterial strains against EOs. Different EOs (as thyme, tea tree, peppermint, clove, lemon, bergamot, lavender, mint), in addition to active EOs components (as carvacrol, thymol and eugenol) have been reported to exert significant antimicrobial activities against different microorganisms such as *Salmonella* spp., *Staphylococcus aureus*, Candida sp*., Listeria monocytogenes.*, *Escherichia coli*, *Klebsiella* sp., *Proteus* sp., and *Pseudomonas aeruginosa* (Ben Hsouna et al., 2017; Çakmak et al., 2020; Ed-Dra et al., 2021; El-Darier et al., 2018; Orlo et al., 2021; Rajkowska et al., 2016; Valková et al., 2021). EOs were initially applied in their free form, nevertheless, they presented several limitations as their strong odors and flavors, low stability, poor water solubility, high volatility, high degradability and low bioavailability that limited their different applications (Radünz et al., 2018). Therefore, recent micro- and nano-encapsulation methods have been developed to overcome these challenges and ensure protection of free EO molecules from the external environmental conditions. In addition, encapsulation has been reported to mask EOs negative organoleptic properties, enhance their antimicrobial activity and ensure a controlled release (da Silva Gündel et al., 2018; Granata et al., 2018; Popiolski et al., 2016; Shetta et al., 2019). Another strategy adopted for the application of EOs was their combination with other hurdles. In most cases, combining EOs with other treatments exerted more pronounced anti-microbial activity and reduced the amounts of EOs being used, thus minimizing production costs, lowering environmental and sensorial impacts, and reducing any potential risk of developing antimicrobial resistance (Duarte et al., 2012; Moosavy et al., 2008; Yamazaki et al., 2004; Yoon et al., 2011).

This review provides an overview of the published data and current knowledge on EOs and their active components as potent antimicrobial tools used to fight different forms of microbial contaminations along with their mechanisms of action. Moreover, this review highlights the strategies used to improve EOs antimicrobial activity by micro- and nano-encapsulation and by their combination with other hurdles.

## 2 Search strategies

In this review, the specialized databases Scopus, ScienceDirect, PubMed and Web of Science were used for the literature search using different combinations of the following keywords: essential oils, antimicrobial, hurdle technology, and encapsulation.

### 2.1 Vegetative cells

Vegetative cells are the most common form of microbial contaminations that are easily detectable and generally inhibited by conventional treatments. Under harsh and stressful conditions as temperature, osmotic stress, desiccation and/or regular cleaning and disinfection procedures, some vegetative cells may adapt by implementing physiological, structural and/or molecular mechanisms making them more resistant (Esbelin et al., 2018; Potts, 2001). Sporulation and biofilm formation are the most common resistance mechanisms developed by microbial cells to survive stressful conditions. In addition, vegetative cells may enter the viable but not culturable physiological state as a response for the stressful conditions.

### 2.2 Viable but non culturable (VBNC) cells

VBNC cells are viable bacterial cells that have low levels of metabolic activity. Several non-spore forming microorganisms and pathogens could enter the VBNC state as *E. coli*, *E. coli* O 157:H7, *L. monocytogenes*, *S. aureus*, *H. pylori*, *P. aeruginosa*, *Salmonella* spp., *Enterococcus faecalis*, Yersinia, *Bacillus coagulans*, *Micrococcus luteus*, *Lactobacillus brevis* and *Vibrio* spp. (Boehnke et al., 2017; Casasola-Rodríguez et al., 2018; Chang and Lin, 2017; Doležalová et al., 2016; Han et al., 2018; Highmore et al., 2018; Jiang et al., 2015; Kibbee and Ormeci, 2017; Liu et al., 2018; Majeed et al., 2018; Mukamolova et al., 2018; Orta et al., 2017; Zhang et al., 2015; Zhao et al., 2016; Zhong and Zhao, 2018). Despite being in a stressed state, VBNC cells maintain a persistent cellular biology and structure with normal gene expression (Fakruddin et al., 2013). However, they are not culturable into colonies on standard bacteriological culture media and are unfortunately not detected by classical microbiological techniques. Alternative microscopic and molecular detection methods are thus required to detect them (Dong et al., 2020). With appropriate conditions, VBNC cells are resuscitated, repaired and become again culturable and metabolically active (Dong et al., 2020). Thus, they remain a public health concern with their ability to cause severe infections and diseases (Schottroff et al., 2018).

### 2.3 Microbial spores

Under unfavorable growth conditions, some microorganisms including mainly Clostridia and Bacilli may form metabolically dormant spores as an adaptive survival strategy (Setlow, 2014). Due to their unique structure and components, spores exhibit a better resistance state than their vegetative forms against several environmental, chemical and physical stresses (Trunet et al., 2017). They are able to escape the destruction by different types of treatments even the most severe ones as mechanical disruption, heating and/or the exposure to a variety of chemicals (Burgess et al., 2010). Spores are able to adhere tightly to both abiotic and biotic surfaces and spread to contaminate other surfaces, individuals or appliances (Mallozzi et al., 2010). Under proper conditions, dormant spores are able to recover by germination and return back into sensitive vegetative cells that are responsible for severe food contaminations, intoxications and illnesses, along with nosocomial infections (Mallozzi et al., 2010; Wells-Bennik et al., 2016). Moreover, microbial cells can communicate through small diffusible chemical signal molecules known as the autoinducers that regulate the quorum sensing (QS) mechanism involved in the coordination of the community activities and in monitoring their population density (Afonso et al., 2020; Palla et al., 2018). QS was shown to regulate spore germination, the metabolite productions as well as the development of an antimicrobial resistance (Narla et al., 2021; Preda and Sãndulescu, 2019). Studies have demonstrated that the inhibition of QS signaling molecules suppressed the initiation of sporulation as well as spores' germination (Vesty et al., 2020; Wang et al., 2018; Xiong et al., 2021).

### 2.4 Molds and mycotoxins

Molds are responsible for the production of prominent amounts of mycotoxins that have become an important global issue. The main mycotoxin producers are *Penicillium*, *Aspergillus*, *Fusarium* and *Alternaria* (Ramirez et al., 2018). The Food and Drug Organization estimates that more than 25% of food produced worldwide are contaminated to a certain level with mycotoxins (Magnoli et al., 2019). Mycotoxins can be responsible for acute and/or chronic toxicity, depending on the type and dose of the toxin, as well as the health and age of the exposed individual or animal. They pose great threats to the quality of food products and to the global food security (Guo et al., 2023; Singh et al., 2021). Moreover, due to their greater stability and as they are unavoidable, their prevention and/or elimination remains very challenging (Guo et al., 2023). Studies have shown that QS mechanisms with a variety of signaling molecules, play a major role in the regulation of molds and mycotoxins processes such as the growth of molds, hyphae formation, and mycotoxins production (Barriuso et al., 2018; Huang et al., 2020). Therefore, convenient control strategies that target QS mechanism should be adopted to control molds and their associated mycotoxins.

### 2.5 Planktonic cells and biofilms

Biofilms are complex communities of single or multiple microbial species able to attach and colonize different types of abiotic and biotic surfaces in food and water processing plants, industrial, clinical and biomedical fields (Bazargani and Rohloff, 2016; Khelissa et al., 2017). If the conditions are favorable, adherent cells to surfaces will multiply and secrete an extracellular matrix of organic polymers in which they become embedded. The matrix acts a protective barrier against different harmful and/or stressful conditions, provides nutrients, favors horizontal gene transfer and metabolic cooperation between bacterial species (Khelissa et al., 2017; Sandasi et al., 2011). Moreover, increased cell densities in biofilm structure, favors the QS signaling that could control tightly the expression of many genes promoting biofilm formation, as well as the transfer of genetic material between cells (Afonso et al., 2020; Li and Tian, 2012; Narla et al., 2021).

Thus, once biofilms are firmly established, it becomes very difficult to remove and control them as microorganisms grow as communities that associate in different layers. Over time, biofilms have shown potent resistance against different conventional commercial disinfectants (as peroxyacetic acid, quaternary ammonium and chlorine compounds) and have become up to 1000 fold more resistant than their planktonic microbial cells (Abdallah et al., 2014). Biofilms resistance is multifactorial and can be linked to the diffusion limitations of antimicrobials, phenotypic adaptations and genetic mutations (Bridier et al., 2011). Therefore they remain an important public health issue as they could disperse and revert into their planktonic form, colonizing thus new habitats and resulting in cross-contaminations, transmission of diseases, reduced products shelf-life and may possibly restart biofilm formation (Donlan, 2002; Srey et al., 2013; Valeriano et al., 2012). Biofilms could be wet or dry in relation to their growth environment. Wet biofilms are formed in hydrated environments whereas, dry biofilms are formed on hard and soft dry environmental surfaces (Almatroudi et al., 2015). Wet biofilms identified on medical devices with the presence of liquid and/or moisture are responsible for 65% of healthcare-associated infections (Ledwoch et al., 2018). Some studies showed that dry biofilms have a more pronounced heat resistance compared to wet biofilms.

For example, upon heating up to 121 °C for 20 min, dry *S. aureus* dry biofilms have shown higher heat resistance than wet biofilms and planktonic cells with a reduction of 2, 7 and 8 logs, respectively (Almatroudi et al., 2018). In fact, dry biofilms acquired a cross protection and an increased ability to withstand extreme conditions as they were exposed to sub-lethal periodic dehydrations that led to osmotic stress (Almatroudi et al., 2018). In another study, *Bacillus cereus* spores formed in dry biofilms on stainless steel and polystyrene surfaces were less heat resistant than spores formed in wet biofilms except for dry spores from *B. cereus* NIZO 4088 (Hayrapetyan et al., 2016). Drying biofilms by air exposure, increased spores formation and accelerated their germination and release from mother cells making them less heat resistant (Hayrapetyan et al., 2016).

## 3 Essential oils and their active components as potent antimicrobials

### 3.1 Essential oils composition

**Figure 1:**
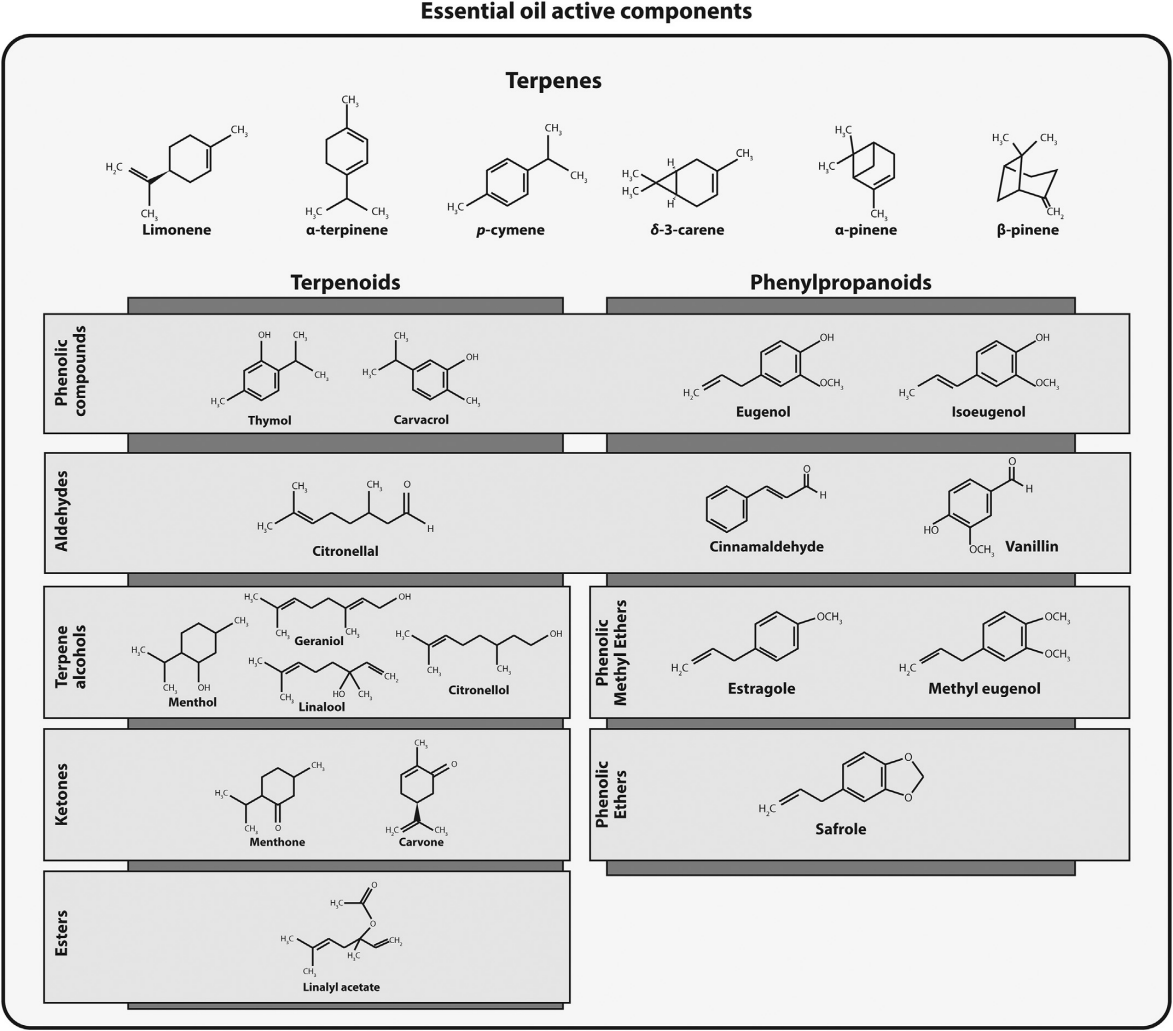
Chemical structure and function of some of the most common essential oils active components.

EOs are natural complex antimicrobials constituted of different major active components that some can be found at relatively high concentrations (between 20 and 70%) and others in trace amounts (Bakkali et al., 2008). The chemical composition and amount of extracted EOs depend on the harvest period, climate, soil type, plant and the extraction technique (García-Salinas et al., 2018). EOs active constituents are generated by different biosynthetic pathways and predict EOs biological properties. The different active compounds can be classified into terpene compounds with their oxidative derivatives terpenoids or into phenylpropanoids along with their aromatic derivatives (Figure 1) (Dhifi et al., 2016). More than 4000 different terpenes and only around 50 phenylpropanoids have been discovered till present (Omonijo et al., 2018).

Terpenes are hydrocarbon compounds of general formula (C_5_H_8_) _n_ where “n” represents the number of isoprene units (5-carbon blocks) (El Asbahani et al., 2015). Depending on “n”, terpenes could be classified as monoterpenes (C_10_H_16_), sesquiterpenes (C_15_H_24_), diterpenes (C_20_H_32_) and triterpenes (C_30_H_40_) (Swamy et al., 2016). Monoterpenes (as *p*-cymene, α-pinene, limonene, β-pinene, α-terpinene and δ-3-carene) constitute 90% of EOs composition but have shown no remarkable antimicrobial activity when used alone as they have a benzene ring with no functional groups on their side chains (Bakkali et al., 2008; Hyldgaard et al., 2012; Dhifi et al., 2016).

The biochemical modifications of terpenes by adding oxygen molecules, and removing or moving methyl groups via enzymes results in the formation of terpenoids (as carvacrol, thymol, citronellal, menthol, linalool, geraniol and linalyl acetate) (Bakkali et al., 2008; Hyldgaard et al., 2012). Terpenoids could be divided according to their functionalities into aldehydes, ketones, phenols, alcohols, esters, ethers, acids and epoxides (Chouhan et al., 2017). Their antimicrobial activity is mainly linked to the presence of functional groups.

Some EOs contain other oxygenated molecules known as phenylpropanoids (Dima and Dima, 2015). They are less frequent than terpenes and the mostly studied ones were eugenol, safrole, isoeugenol, cinnamaldehyde and vanillin (Hyldgaard et al., 2012). Aromatic compounds derived from phenylpropanoids may comprise phenolic acids, aldehydes, alcohols, ketones, esters, amines, epoxides and sulfides (Calo et al., 2015).

The greatest antimicrobial activity was reported for phenolic compounds mainly carvacrol, thymol and eugenol (Al-Maqtari et al., 2021; Tariq et al., 2019). This is related to the presence of an acidic hydroxyl group and delocalized electrons, followed by aldehydes (cinnamaldehyde, citronellal), terpene alcohols (geraniol, citronellol, menthol, linalool, α-terpineol, terpinen-4-ol) and then EOs containing esters (cedryl acetate) or ketones (menthone, carvone, camphor, thujone) (Al-Maqtari et al., 2021; Dhifi et al., 2016; Hyldgaard et al., 2012; Kalemba and Kunicka, 2003; Omonijo et al., 2018).

### 3.2 Antimicrobial mechanisms of essential oils and their active components

The activity of EOs and their active components has been reported against several types of bacteria, yeasts, molds and was found to be related to EOs chemical composition and structure, in addition to the type and structure of targeted microorganisms (Al-Maqtari et al., 2021; Kalagatur et al., 2018).

Against yeasts and molds, EOs were found to disrupt their membranes and damage their endomembrane system (Trindade et al., 2015; Hu et al., 2019). Additionally, EOs were reported to interfere with biofilms 3D structure reducing extracellular polymeric substances, to inhibit biofilms metabolic activity and respiration with subsequent adverse effects on mitochondria (Almeida et al., 2016; Braga et al., 2008; Hu et al., 2019).

Most studies reported that EOs are slightly more efficient against Gram-positive bacteria than Gram-negative bacteria (Brenes and Roura, 2010; Vidács et al., 2018; Aiemsaard et al., 2011; Bazargani and Rohloff, 2016; de Medeiros Barbosa et al., 2016). The presence of lipophilic ends of lipoteichoic acid in cell membranes of Gram-positive bacteria makes them more susceptible for EOs penetration (Chouhan et al., 2017). Moreover, Gram-negative bacteria are more resistant to the action of EOs due to their rigid and complex outer membrane rich in lipopolysaccharides providing a barrier and restricting the penetration of hydrophobic molecules (Bhargava et al., 2015; Bazargani and Rohloff, 2016). However, some studies reported that Gram negative bacteria are more sensitive to EOs than Gram positives. For example, Gram-negative such as *Acinetobacter baumannii* and *Vibrio parahaemolyticus* were the most sensitive microorganisms to *Eucalyptus camaldulensis* EO (Aleksic Sabo and Knezevic, 2019). Similarly, Gram-negative enteropathogenic *E. coli* biofilms were more sensitive than Gram-positive *L. monocytogenes* sessile cells to cinnamon EO and cinnamaldehyde (de Oliveira et al., 2012). In addition, it was reported that Gram-negative *Salmonella* were found more susceptible to both oregano and thyme EOs than *L. monocytogenes* (El Moussaoui et al., 2013). Studies have also shown that different strains of the same bacterium may exert highly variable responses to EOs (Sales et al., 2017; Marotta et al., 2016). It remains thus of utmost importance to evaluate the antimicrobial activity of EOs and their active components against different bacteria as well as against different strains of the same bacterium.

**Figure 2:**
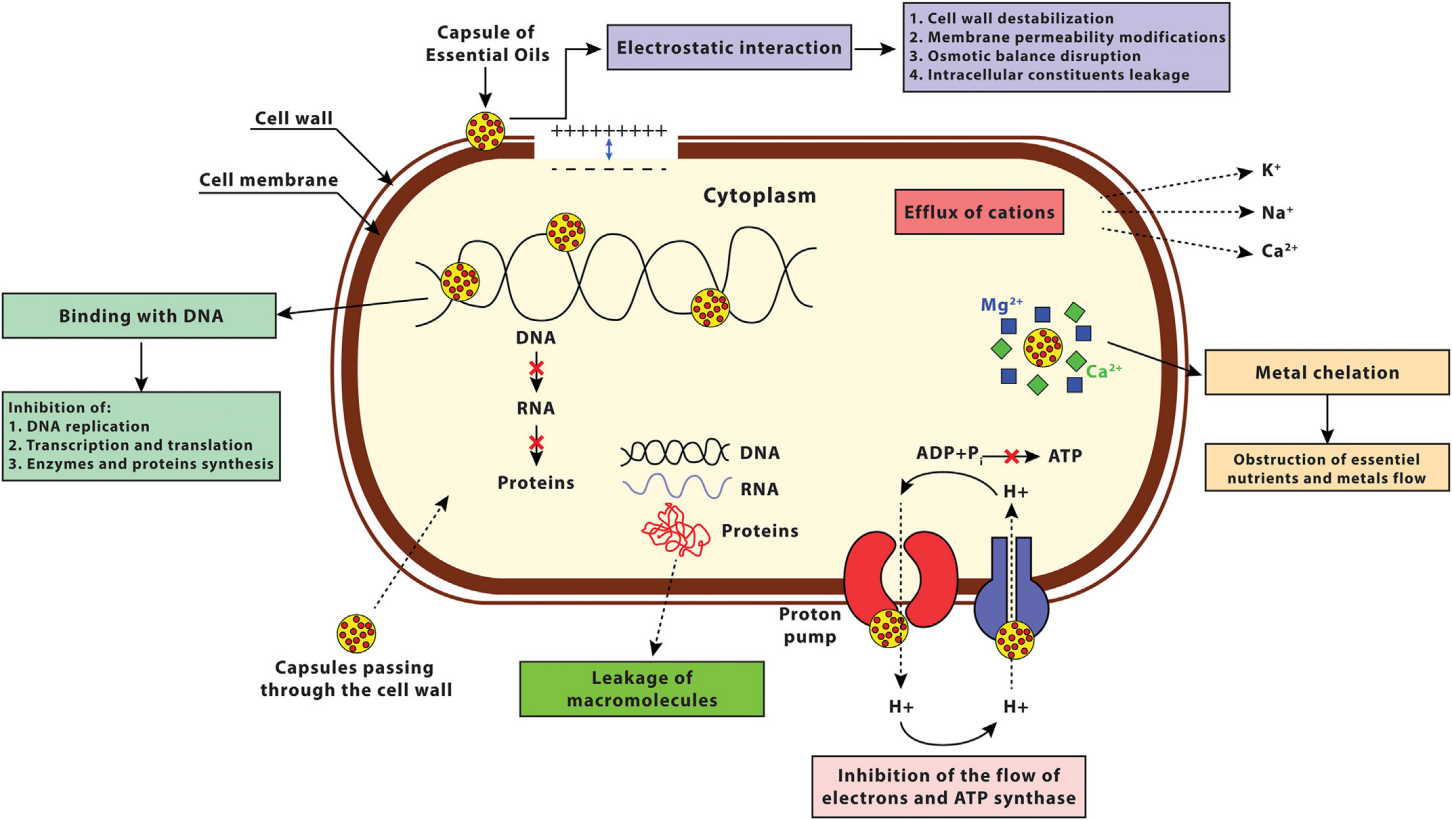
Different antimicrobial mechanisms of action of encapsulated essential oils.

Depending on their chemical composition and structure, different mechanisms have been proposed for the antimicrobial activity of EOs and their active components (Figure 2). Originally, their mechanisms of action were limited to the destruction of cells cytoplasmic membranes, but additional mechanisms have been proposed recently.

The mechanism of action of several EOs and their phenolic compounds was mainly linked to their attachment and destabilization of bacterial cellular membranes. Owing to their lipophilic nature, EOs and their active components were reported to penetrate and accumulate easily in the lipid bilayer of cytoplasmic membranes. They align between the fatty acid chains of cell membranes causing destabilization and degradation of its different layers through an inactivation of enzymatic mechanisms or a destruction of electron transport system (Bhavaniramya et al., 2019; Burt, 2004; Dhifi et al., 2016; de Oliveira et al., 2010; Swamy et al., 2016). Subsequently, this leads to the breakdown of the integrity of the phospholipid bilayer and an increase in its permeability. The enhanced permeability favors the penetration of antimicrobial agents, disrupts the normal cellular function and results in a leakage of the vital intracellular content (ions, proteins, nucleic acids), a disruption of the proton motive force (PMF), a reduction in the membrane potential and a depletion of adenosine triphosphate (ATP) (Burt, 2004; Pathania et al., 2018; Zhang et al., 2017). The loss of intracellular components is tolerated to a certain amount with no loss of viability (Ju et al., 2019). However, the greater the contact time between EOs and microorganisms the higher is the loss of intracellular content that further leads to cell death by necrosis or apoptosis (Bakkali et al., 2008; Valeriano et al., 2012).

In literature studies, *Salvia sclarea* EO, carvacrol and eugenol were able to disrupt the membrane integrity and increase the permeability of *L. monocytogenes*, *S. aureus* and *E. coli* cellular membranes (Gill and Holley, 2006; Khan et al., 2017; Cui et al., 2015). This was confirmed by the dissipation of PMF, release of DNA and proteins, and the decrease in intracellular ATP with subsequent increase in extracellular ATP (Gill and Holley, 2006; Khan et al., 2017; Cui et al., 2015). The permeability of *E. coli* and *S. aureus* membranes was also altered after exposure to terpenes and phenylpropanoids. This alteration in permeability allows the relatively large molecules to pass easily through the membrane (Nogueira et al., 2021). Exposure of methicillin-resistant *S. aureus* (MRSA), *E. coli* and *B. subtilis* to *Amomum villosum* Lour and *Origanum compactum* EOs induced a leakage of macromolecular molecules such as DNA, RNA and proteins (Bouyahya et al., 2019; Tang et al., 2020). The leakage of vital intracellular constituents such as ions and proteins from *S*. Enteritidis and *E. coli* cells was also reported after exposure to carvacrol and thymol (Yammine et al., 2022). Also, the application of 0.2% v/v mustard EO caused a significant loss of intracellular ATP of both *E. coli* O157:H7 and *Salmonella typhi* (Turgis et al., 2009). Similar results were reported when applying 0.006–0.025% oregano EO against *L. monocytogenes* and *E. coli* 0157:H7 (Caillet et al., 2005; Caillet and Lacroix, 2006). Following treatment with carvacrol, oregano and eugenol, leakages of phosphate and potassium ions were reported from *S. aureus*, *P. aeruginosa* and *E. coli* bacterial membranes (Lambert et al., 2001; Walsh et al., 2003; Bouhdid et al., 2009). In Gram-negative bacteria, carvacrol and thymol have been reported to break down the outer membrane, releasing lipopolysaccharides and thus destabilizing and increasing the permeability of cytoplasmic membranes with loss of ATP (Pérez-Conesa et al., 2011; Turgis et al., 2009). Moreover, other studies reported that sub-inhibitory concentrations of citral and *Thymus vulgaris* EOs provoked increased sub-lethally injured *Listeria* and *Salmonella* bacterial cells, respectively (Ed-Dra et al., 2021; Silva-Angulo et al., 2015). Injured bacterial cells had a higher sensitivity to unfavorable conditions enhancing thus the effectiveness of other preservation methods added as salt.

Other mechanisms of action have been proposed for EOs phenolic compounds as their interference with specific proteins and their inhibition of flagella and toxins synthesis. An overnight exposure of *E. coli* O157:H7 to 1 mM (approximately 0.015%) carvacrol induced significant production of heat shock proteins 60 (which are usually secreted by bacteria under stressful and toxic conditions) and inhibited significantly the synthesis of flagella (Burt et al., 2007). Another effect reported of EOs on cell membranes was the inhibition of toxins production as the case reported when carvacrol and oregano inhibited the release of *B. cereus* and *S. aureus* toxins, respectively (Ultee and Smid, 2001). The mechanism of action to limit toxins production was probably related to the insufficient PMF or ATP energy to export the secreted toxins out of the bacterial cells or to the little energy left for toxins production as cells conserve the available energy for survival functions (Ultee and Smid, 2001). It has been also established that eugenol inhibited the activity of several enzymes such as amylase, ATPase, protease and histidine decarboxylase (Hyldgaard et al., 2012). An additional mechanism of action has been proposed for carvacrol that acted as a transmembrane carrier due to the presence of phenolic hydroxyl group and a system of delocalized electrons (Ultee et al., 2002). Carvacrol carried its hydroxyl group into the cytoplasm through the cytoplasmic membrane and carried back out a cation (mainly potassium) from the cytoplasm to the external environment (Hyldgaard et al., 2012).

Additionally, some phenolic compounds and aldehydes have shown the ability to suppress the expression of bacterial genes related to biofilm formation, to interfere with (QS) signaling and to prevent cell division and thus lead to cell death without causing a damage to bacterial cell membranes (Amalaradjou and Venkitanarayanan, 2011; Brackman et al., 2008; Domadia et al., 2007; Kwon et al., 2003). EOs and their active components have shown to breakdown the QS communication mechanism by interacting with bacterial cell wall receptors, reducing thus the reception of signaling molecules and degrading this cell-to-cell communication that eventually inhibits biofilms formation (Maurya et al., 2021). Luciardi et al. (2016) reported an inhibitory effect of *Citrus reticulata* EO on QS signaling and on the production of autoinducers and elastase enzymes that subsequently inhibited the formation of *P. aeruginosa* biofilms. Epigallocatechin gallate, a polyphenol found in tea extracts, was also found to down regulate a quorum sensing gene (*agr*A) in *L. monocytogenes* biofilms (Du et al., 2018). Cinnamaldehydes which are considered the aldehydes with the strongest antimicrobial activity, were able in different studies to bind to the FtsZ cell division regulating protein, inhibiting thus the process of cell division (Domadia et al., 2007; Hemaiswarya et al., 2011). Also, aldehydes functional groups were found to bind covalently with DNA of bacterial cells, thereby interfering with the normal translation and transcription functions with a subsequent blockage of proteins synthesis (Hyldgaard et al., 2012).

### 3.3 Free essential oils versus their active components

Several free EOs and their active components have been screened for their potent antimicrobial activity against a broad range of microorganisms. They were reported to induce significant reductions or inhibitions of microbial contaminations (Adukwu et al., 2012; Almeida et al., 2016; Amaral et al., 2015; Bazargani and Rohloff, 2016; Braga et al., 2008; de Medeiros Barbosa et al., 2016; García-Salinas et al., 2018; Kwieciński et al., 2009; Rattanachaikunsopon and Phumkhachorn, 2010; Scotti et al., 2021), as well as inhibitions of spore germination, mycelial growth and mycotoxins secretion (Hu et al., 2019; Xu et al., 2021) (Table 1). However, the use of EO as a whole or using its active major components alone to control microbial contaminations is still a debatable issue.

**Table 1:** In vitro antimicrobial activity of free EOs and their active components against different types of microbial contaminations.

Target microorganisms	EOs and active components	MIC^a^	Exposure concentration, time	Antimicrobial activity	References
Gram-positive bacteria					
*S. aureus*	*Cymbopogon flexuosus*	0.06%	0.03–4%, 24 h	Complete inhibition at MIC	Adukwu et al. (2012)
	*Origanum compactum*	0.031–1%	MIC and 1.5MIC, 0–120 min	Immediately significant reductions at 1-1.5MIC	Bouhdid et al. (2009)
	*Melaleuca alternifolia*	0.12–0.50%	0–8%, 15–60 min	Significant reduction with 0.5% EO after 60 min	Kwieciński et al. (2009)
	*Origanum vulgare* L., Carvacrol	2.5–5 μl/ml	0.31–2.5 μl/ml, 24 h	No decrease in microbial counts	dos Santos Rodrigues et al. (2017)
	Carvacrol, CIN, Thymol	0.2–0.4 mg/ml	0.01–4 mg/ml, 24 h	Significant inhibition at MIC Around 3–5 log reductions with 0.5–1 mg/ml EO	García-Salinas et al. (2018)
	*Salvia officinalis*, *Mentha spicata*	1.25%	2.5–5%, 6 min	2.3–3.1 log reductions	Vetas et al. (2017)
*S. aureus* (Biofilms)	*Cymbopogon flexuosus*	0.06–0.12%	0.06–4%, 24 h	Prevention of biofilm formation at MIC and total loss of viability at 0.125–4%	Adukwu et al. (2012)
	*Melaleuca alternifolia*	0.12–0.50%	0–8%, 0–120 min	Complete eradication and inhibition of metabolism with 1% EO after 120 min	Kwieciński et al. (2009)
	*Origanum vulgare* L., Carvacrol	5–10 μl/ml	0.31–2.5 μl/ml, 360 h	Decrease in counts after 216 h exposure to 1.25–2.5 μl/ml	dos Santos Rodrigues et al. (2017)
	Carvacrol, CIN, Thymol	N/A	0.25–1 mg/ml, 24 h	Significant inhibition at MIC Around 3–5 log reductions with 0.5–1 mg/ml EO	García-Salinas et al. (2018)
	*Origanum vulgare* L.	10 μl/ml	5–10 μl/ml, 10–15 min	Complete removal of *S. aureus* LPMA63 but not *S. aureus* LPMA11 biofilms	Dos Santos Rodrigues et al. (2018)
	Carvacrol	5 μl/ml	2.5–5 μl/ml, 10–15 min	Complete removal of all biofilms	
	*Salvia officinalis*, *Mentha spicata*	0.63–1.25%	5–15%, 6 min	0.8–3 log reductions	Vetas et al. (2017)
*S. epidermidis* spp.	*Mutellina purpurea,* *α-pinene*	0.31–0.625 mg/ml	0.15–2.5 mg/ml, 24 h	Complete inhibition at 0.312–0.625 mg/ml	Sieniawska et al. (2013)
	*Cinnamomum burmannii*	0.5–1%	MIC, 24 h	Complete inhibition	Nuryastuti et al. (2009)
*S. epidermidis* spp. (Biofilms)	*Mutellina purpurea,* *α-pinene*	0.62–1.25 mg/ml	2.5–10 mg/ml, 24 h	Concentrations >10 mg/ml were needed to eradicate biofilms	Sieniawska et al. (2013)
	*Cinnamomum burmannii*	1–2%	0.01–2%, 1–24 h	Complete loss of metabolic activity after 3–24 h exposure with detachment of biofilms	Nuryastuti et al. (2009)
*Enterococcus* spp.	*Cymbopogon flexuosus*, *Thymus vulgaris*	0.47–15 mg/ml	MIC, 24 h	Complete inhibition	Quendera et al. (2019)
*Enterococcus* spp., (Biofilms)	*Cymbopogon flexuosus*, *Thymus vulgaris*	N/A	1.9–30 mg/ml, 30 min-1 h	Higher levels of eradication were reported for *Aeromonas* spp. at 15 and 30 mg/ml for all treatment times	
*L. monocytogenes*	*Corydothymus capitatus*	0.02%	0.06–0.02%, N/A	At 0.025%, significant effect on murein composition and reduction of intracellular ATP with an increase in extracellular ATP	Caillet et al. (2005); Caillet and Lacroix (2006)
	*Origanum vulgare* L., *Rosmarinus officinalis* L.	0.6–10 μl/ml	MIC, 24 h	Significant reduction after 2–8 h exposure	de Medeiros Barbosa et al. (2016)
*L. monocytogenes,* (Biofilms)	Thyme, Tea tree	0.01–0.09%	MIC and 0.1%, 2 h	Significant 1.4–3.3 log reductions of sessile cells when treated with MIC and 0.1%.	Sadekuzzaman et al. (2018)
	Thyme, Oregano, Carvacrol	N/A	0.10–0.50%, 24h	Complete inactivation with 0.25% for 24 h	Desai et al. (2012)
**Gram-negative bacteria**					
*E. coli*	Eugenol, CIN^b^	1 μg/ml	0.5–2 μg/ml, 0–90 min	Complete inhibition at MIC after 60–75 min	Ali et al. (2005)
	Carvacrol, Thymol	200 mg/l	MIC, 12 h	No complete inhibition but significant reductions	Xu et al. (2008)
	Carvacrol, CIN, Thymol	0.2–0.4 mg/ml	0.01–4 mg/ml, 24h	Significant inhibition at MIC Around 3–5 log reductions with 0.5–1 mg/ml EO	García-Salinas et al. (2018)
	Black pepper	1.0 μl/ml	MIC and 2MIC, 24 h	Significant inhibitory effect of 96.73% after 24 h exposure to 2MIC	Zhang et al. (2017)
	*Origanum vulgare* L., *Rosmarinus officinalis* L.	0.6–10 μl/ml	MIC, 24 h	Significant reduction after 2–8 h exposure	de Medeiros Barbosa et al. (2016)
	*Thymbra capitata*	0.05%	1%, 30–60 s	Inhibition of approximately 8 logs after 30–60 s	Falcó et al. (2019)
*E. coli*, (Biofilms)	*Thymbra capitata*	N/A	0.25–2.5%, 1–10 min	Reduction of >3 logs after 10 min exposure to 2.5%	Falcó et al. (2019)
*E. coli* O157:H7	Carvacrol	N/A	1 mM = 0.015%, overnight	Significant inhibition of flagella synthesis and production of heat shock proteins 60	Burt et al. (2007)
	*p*-cymene	N/A	1–10 Mm, overnight	No significant activity	
	*Corydothymus capitatus*	0.02%	0.06–0.02%, N/A	At 0.025%, significant effect on murein composition and reduction of intracellular ATP with an increase in extracellular ATP	Caillet et al. (2005); Caillet and Lacroix (2006)
	Mustard	0.2%	0–0.2%, 30 min	At 0.1 and 0.2%, a significant increase in both extracellular ATP and cell constituents' release.	Turgis et al. (2009)
*E. coli* O157:H7 (Biofilms)	Thyme, Tea tree	0.01–0.09%	MIC and 0.1%, 2 h	Significant 1.4–3.3 log reductions of sessile cells when treated with MIC and 0.1%.	Sadekuzzaman et al. (2018)
*Salmonella* spp.	*Origanum vulgare* L., *Rosmarinus officinalis* L.	0.6–10 μl/ml	MIC, 24 h	Significant reduction after 2–8 h exposure	de Medeiros Barbosa et al. (2016)
	*Thymbra capitata*	0.05%	1%, 30–60 s	Inhibition of approximately 8 logs after 30–60 s	Falcó et al. (2019)
	Mustard	0.2%	0–0.2%, 30 min	At 0.1 and 0.2%, a significant increase in both extracellular ATP and cell constituents' release.	Turgis et al. (2009)
	Thyme, Oregano, Carvacrol	0.025%	0.006–0.1%, 24 h	Complete inhibition at MIC	Soni et al. (2013)
*Salmonella* spp. (Biofilms)	*Thymbra capitata*	N/A	0.25–2.5%, 1–10 min	Around 1 log reduction after 10 min exposure to 2.5%	Falcó et al. (2019)
	Carvacrol, Thymol	156–312 μg/ml	16–624 μg/ml, 1 h	Reduced established biofilms but no complete inhibition	Amaral et al. (2015)
	Thyme, Tea tree	0.01–0.09%	MIC and 0.1%, 2 h	Significant 1.4–3.3 log reductions of sessile cells when treated with MIC and 0.1%.	Sadekuzzaman et al. (2018)
	Thyme, Oregano, Carvacrol	N/A	0.006–0.1%, 24 h	Significant reduction after 1 h exposure to 0.05 or 0.1%	Soni et al. (2013)
	*Mentha piperita*, *Cymbopogon citratus*	7.8 μl/ml	MIC, 10–40 min	Significant reductions of 4.03–4.20 logs after 10 min contact and complete inhibition after 20 min	Valeriano et al. (2012)
*P. aeruginosa*	*Origanum compactum*	0.031–1%	MIC and 1.5MIC, 0–120 min	Immediately significant reductions at 1-1.5MIC	Bouhdid et al. (2009)
*P. aeruginosa* (Biofilms)	Carvacrol, Thymol	0.02–0.05%	1/16 –2MIC, 24 h	Inhibited up to 97% at 2MIC	El Abed et al., 2011
*Aeromonas* spp.	*Cymbopogon flexuosus*, *Thymus vulgaris*	0.47–15 mg/ml	MIC, 24 h	Complete inhibition	Quendera et al. (2019)
*Aeromonas* spp. (Biofilms)	*Cymbopogon flexuosus*, *Thymus vulgaris*	N/A	1.9–30 mg/ml, 30 min-1 h	Higher levels of eradication were reported for *Aeromonas* spp. at 15 and 30 mg/ml for all treatment times	Quendera et al. (2019)
	*Thymus vulgaris*, *Cymbopogon citratus*	31–62 μl/ml	MIC, 15 min	Significant reduction of 3.84–4.51 log CFU/cm^2^	Millezi et al. (2013)
**Fungi**					
*C. albicans*	*Cymbopogon winterianus*, *Cinnamon cassia*	65.5–250 μg/ml	7.8–1000 μg/ml, 24–48 h	Complete inhibition at MIC	Almeida et al. (2016)
*C. albicans* (Biofilms)		N/A	1 mg/ml,0–48 h	Significant reductions but no complete inhibition and no prevention of biofilm regrowth	
	Thymol	125 μg/ml	1/8MIC-MIC, up to 24 h	Significant reductions at MIC after 1 h and almost a complete destruction of 3D structure	Braga et al. (2008)

a MIC: Minimum Inhibitory Concentration.

b CIN: Cinnamaldehyde.

Few studies reported a better antimicrobial activity of whole EOs as lemongrass, *Cymbopogon nardus* and cinnamon compared to that of their major components used alone (Trindade et al., 2015; Chang et al., 2001; Aiemsaard et al., 2011) (Table 2). This was mainly explained by the interaction between the different components present in EOs that may have a synergistic or additive antimicrobial activity (Bazargani and Rohloff, 2016; Marino et al., 2001). In these cases, active components (citronellal, citral, geraniol and cinnamaldehyde) were mainly terpenoids with alcohol and aldehyde functions, which explains their weak antimicrobial activity when used alone. In addition, myrcene active component belonging to the terpene group possessed no antimicrobial activity when used alone against several microorganisms in comparison to the whole EO lemongrass (Aiemsaard et al., 2011).

**Table 2:** Comparison of MIC values between free EOs and their active components against different types of microbial contaminations.

Microorganism	Whole EO		EO Active Components		References
	Type	MIC^a^	Type	MIC	
Gram-positive bacteria					
*S. aureus*	Oregano	0.06–0.12%^b^	Carvacrol Thymol	0.01–0.03% 0.03–0.06%	Nostro et al. (2007)
	Oregano	5 μl/ml	Carvacrol	2.5 μl/ml	dos Santos Rodrigues et al. (2017)
	Oregano	575 mg/l	Carvacrol Thymol	175 mg/l 140 mg/l	Lambert et al. (2001)
	Lemongrass	0.54 μl/ml	Citral Geraniol Myrcene	0.62–1.25 μl/ml^c^ 0.62–1.25 μl/ml >17.5 μl/ml	Aiemsaard et al. (2011)
*S. aureus* (Biofilms)	Oregano	0.25–0.50%	Carvacrol Thymol	0.03–0.12% 0.06–0.12%	Nostro et al. (2007)
	Oregano	10 μl/ml	Carvacrol	5 μl/ml	dos Santos Rodrigues et al. (2017)
*S. epidermidis*	Oregano	0.06–0.12%	Carvacrol Thymol	0.01–0.03% 0.03–0.06%	Nostro et al. (2007)
*S. epidermidis* (Biofilms)	Oregano	0.12–0.50%	Carvacrol Thymol	0.03–0.12% 0.03–0.12%	Nostro et al. (2007)
*L. monocytogenes*	Oregano Thyme	0.05% 0.05%	Carvacrol	0.05%	Desai et al. (2012)
*Streptococcus agalactiae*	Lemongrass	0.27–0.54 μl/ml	Citral Geraniol Myrcene	0.31–0.62 μl/ml 0.31 μl/ml >17.5 μl/ml	Aiemsaard et al. (2011)
**Gram-negative bacteria**					
*E. coli*	Thyme	1.60 mg/ml	Carvacrol Thymol	0.20 mg/ml 0.20 mg/ml	Vidács et al., (2018)
	Cinnamon	0.40 mg/ml	CIN^d^	0.20 mg/ml	
	Cinnamon	250 μg/ml	CIN^d^	500 μg/ml	Chang et al. (2001)
	Lemongrass	0.54–1.09 μl/ml	Citral Geraniol Myrcene	1.25–2.5 μl/ml 1.25–2.5 μl/ml >17.5 μl/ml	Aiemsaard et al. (2011)
*S.* Typhimurium	Oregano Thyme	0.05–0.10% 0.05–0.10%	Carvacrol	0.02%	Soni et al. (2013)
*P. aeruginosa*	Oregano	1648 mg/l	Carvacrol Thymol	450 mg/l 385 mg/l	Lambert et al. (2001)
	Cinnamon	250 μg/ml	CIN^d^	1000 μg/ml	Chang et al. (2001)
*K. pneumoniae*	Cinnamon	500 μg/ml	CIN^d^	1000 μg/ml	Chang et al. (2001)
*B. cereus*	Lemongrass	0.13 μl/ml	Citral Geraniol Myrcene	0.15 μl/ml 0.31 μl/ml >17.5 μl/ml	Aiemsaard et al. (2011)
**Fungi**					
*C. albicans*	*C. nardus*	64 μg/ml	Citronellal	512 μg/ml	Trindade et al. (2015)

a MIC: Minimum Inhibitory Concentration.

b MIC values were determined in a range as they were tested on several strains.

c MIC values were determined in a range as the experiments were done in triplicates.

d CIN: Cinnamaldehyde.

Whereas, most of the other studies reported that active EO components had a similar or better antimicrobial activity against different types of microorganisms when compared to the activity of the whole EO (Desai et al., 2012; dos Santos Rodrigues et al., 2017; Nostro et al., 2007; Soni et al., 2013; Vidács et al., 2018). This was related to the presence of some active components, mainly phenolic compounds, such as carvacrol and thymol that form a fraction of EO but are mainly responsible for the wide and strong antimicrobial activity of the whole EO. Thus, higher amounts of EO will be required to exert the same antimicrobial activity as that of the main active component used alone against different types of microbial cells (dos Santos Rodrigues et al., 2017). The lower concentrations of active components used will also reduce the cost, potential toxicity and negative impacts of EOs on the sensory attributes of food products (Calo et al., 2015). Additionally, more reproducible and accurate standardization may be achieved when using active components alone as many factors could affect the chemical composition of whole EOs (de Oliveira et al., 2012).

All tested free EOs and their active components were effective against planktonic bacterial cells, except in two studies where *p*-cymene, oregano and carvacrol did not show significant antimicrobial activity against *E. coli* O157:H7 and *S. aureus* (Burt et al., 2007; dos Santos Rodrigues et al., 2017). Compared to their planktonic counterparts, biofilms required much higher concentrations of EOs or their active components to reach equivalent microbial reductions or not even (Adukwu et al., 2012; Almeida et al., 2016; Karpanen et al., 2008; Kwieciński et al., 2009; Quendera et al., 2019; Sieniawska et al., 2013; Somrani et al., 2021). In several studies, there was no complete eradication of biofilms even when EOs or their active components were used at 2 times their minimum inhibitory concentrations (MIC) determined on planktonic cells or even at higher concentrations (Almeida et al., 2016; El Abed et al., 2011; Falcó et al., 2019; Jadhav et al., 2013; Sandasi et al., 2011; Vetas et al., 2017; Vidács et al., 2018; Amaral et al., 2015; Quendera et al., 2019). Additionally, biofilms grown for longer time required higher concentrations of EOs and prolonged exposure time to be eliminated (Desai et al., 2012; Braga et al., 2008).

The intrinsic composition of food may influence as well the bacterial sensitivity and decrease EOs antimicrobial activity. It has been found that greater concentrations of free EO or their active components were needed to achieve an equivalent antimicrobial effect in food when compared to in vitro assays due to the interaction of EOs with food components (Cava et al., 2007; Gutierrez et al., 2008; Rattanachaikunsopon and Phumkhachorn, 2010; Singh et al., 2003). This may develop unpleasant organoleptic impacts and decrease the overall acceptability of food products (Jiménez et al., 2018; Radünz et al., 2018). EOs or their active components were found to be more effective in zero or low fat food compared to food with high fat levels (Rattanachaikunsopon and Phumkhachorn, 2010; Cava et al., 2007; Singh et al., 2003). Also, the antimicrobial activity of EOs may be related to some external determinants as pH, temperature and oxygen presence. Generally, at low pH, the antimicrobial activity of several EOs was enhanced due to their increased hydrophobicity and easier dissolution in bacterial cell membranes (Ali et al., 2005; Cava et al., 2007; Gutierrez et al., 2008; Nostro et al., 2012). The temperature effect on the antimicrobial activity of EOs was contradictory in some studies. Lower temperatures (7 °C compared to 35 °C) were found to enhance the antimicrobial activity of EOs or their active components due to the increase in unsaturated phospholipids in cytoplasmic membranes composition with a subsequent increase in membranes' fluidity (Cava et al., 2007). The increased fluidity weakens the membrane attachment and enables an easier dissolution of EOs into it (Cava et al., 2007). On the contrary, the antimicrobial activity of carvacrol combined with cymene was found better at 25 °C compared to their activities at 15 and 4 °C (Rattanachaikunsopon and Phumkhachorn, 2010). There was no clear explanation for the decreased sensitivity of bacterial cells to antimicrobial agents at lower temperatures but it could be attributed to changes in membrane’s properties and/or fluidity, or to low temperatures that may affect the synthesis of target sites in both EOs and bacterial cytoplasmic membranes which affects the sensitivity of microorganisms to EOs (Rattanachaikunsopon and Phumkhachorn, 2010). Also, the antimicrobial activity of EOs was found to be higher in the presence of low oxygen (vacuum packaging and modified atmosphere packaging) as compared to aerobic conditions (Skandamis et al., 2002; Tsigarida and Nychas, 2001).

In most published studies, the required time to eliminate planktonic or sessile cells by free EOs was between 1 and 216 h, which was considered to be neither practical nor economical for industrial applications. For example, at MIC concentrations, oregano, *Cymbopogon nardus*, eugenol and cinnamaldehyde required 2–8 h, 1 h, 9 h and 12 h to reduce significantly (up to 6 log reductions) planktonic and sessile microorganisms of *L. monocytogenes*, *E. coli*, *S. enterica* and *H. pylori*, respectively (Ali et al., 2005; de Medeiros Barbosa et al., 2016; de Oliveira et al., 2010). In other studies, higher concentrations than MIC detected on planktonic cells and a prolonged exposure time were required to induce significant reductions*.* In lab cultures, *Cymbopogon flexuosus* and *Thymus vulgaris*, thyme, oregano and carvacrol, and black pepper were needed at least at 2 MIC concentrations for a minimum of 1 h to induce significant reductions (up to 7 logs) in *Aeromonas* spp. and *Enterococcus* spp., *Salmonella* and *E. coli* (Quendera et al., 2019; Soni et al., 2013; Zhang et al., 2017). In few other studies, lower exposure times (10–20 min) to 5–10 μl of oregano and carvacrol (dos Santos Rodrigues et al., 2018), and to the MIC of peppermint and lemongrass (Valeriano et al., 2012) reduced significantly *S. aureus* and *S. enterica* sessile cells, respectively. *Thymbra capitata* was the only EO reported to induce more than 8 log reductions in both *E. coli* and *S. enterica* in maximum 60 s, but with 20 times its MIC value (Falcó et al., 2019).

### 3.4 Essential oils and their active components versus synthetic antimicrobials

In recent years, EOs and their active components have received particular attention and emerged as effective biosourced candidates to fight the different forms of microbial contaminations and replace the synthetic antimicrobials. Beside the numerous advantages of EOs, many studies showed that their antimicrobial activity could be even better or equivalent to that of synthetic antimicrobials (dos Santos Rodrigues et al., 2018; Vidács et al., 2018). For example, no sessile (<1 log CFU/cm^2^) *S. aureus* LPMA63 biofilms were detected on stainless steel after 15 min exposure to EOs active components oregano (10 μl/ml) and carvacrol (5 μl/ml), whereas 15 min exposure to 250 mg/L of a synthetic antimicrobial (sodium hypochlorite) was ineffective to eliminate biofilms and induced approximately 3.5 log CFU/cm^2^ reductions because of its limited diffusion (dos Santos Rodrigues et al., 2018). Cinnamon, thyme and marjoram EOs showed to have equivalent or better antimicrobial effect against *E. coli* and *L. monocytogenes* biofilms compared to chemical sanitizers conventionally used in food industries such as peracetic acid and sodium hypochlorite (Vidács et al., 2018). Also, in a similar study, cinnamon EO and cinnamaldehyde proved to have similar or greater antimicrobial activity against enteropathogenic *E. coli* (EPEC) and *L. monocytogenes* when compared to commercial synthetic sanitizers such as alkyl dimethyl benzyl ammonium chloride (ADBAC), sodium hypochlorite and hydrogen peroxide (de Oliveira et al., 2012).

A great number of synthetic antimicrobials has been widely applied to encounter microbial contaminations. Nevertheless, the long term exposure to sub-inhibitory concentrations of these antimicrobials induced microbial resistance and thus reduced their effectiveness (El Sayed Zaki et al., 2019). It was reported that 22 % of *L. monocytogenes* strains were resistant and 30% of *S. aureus* isolates have shown reduced susceptibility towards benzalkonium chloride (BAC) (Jiang et al., 2016; El Sayed Zaki et al., 2019). In another study, *L. monocytogenes* harboring quaternary ammonium compounds (QACs) resistance genes (qacH and bcrABC) were found to be prevalent in food processing environments (Møretrø et al., 2017). Several sanitizers such as acetic acid, NaOH, Na_2_SO_4_, glyceryl monolaurate, BAC and peracetic acid were ineffective to eliminate totally *L. monocytogenes* and *S. aureus* planktonic and sessile cells (Chavant et al., 2004; Ibusquiza et al., 2011; Khelissa et al., 2019). Synthetic antimicrobials were also reported to be ineffective in the total elimination of biofilms. An exposure for 10 min to sodium hypochlorite (1000–20,000 ppm) did not eliminate totally *S. aureus* dry biofilms (Charlebois et al., 2017). Also, *L. monocytogenes* biofilms showed resistance to chlorine followed by peroxyacetic acid and QACs (Belessi et al., 2011). *C. perfringens* biofilms were reported to be resistant to disinfectants commonly used in food processing environment and in farms like quaternary ammonium chloride, sodium hypochlorite, potassium monopersulfate, glutaraldehyde and hydrogen peroxide (Charlebois et al., 2017). QACs were very effective to kill more than 98% of *L. monocytogenes* planktonic cells but ineffective against 7 days old biofilms (Chavant et al., 2004).

Additionally, a potential risk of exposure to QAC disinfectants was reported as they are discharged from several sources and eventually reach freshwater and marine environments, soils, sediments and wastewater systems (Mulder et al., 2018). Despite being generally biodegradable under aerobic conditions, QACs adsorption into environmental surfaces and organisms cell membranes is faster than their degradation as they have long half-life degradation (Zhang et al., 2015). Thus, long-term exposure to residual QACs was found to have a great toxic effect not only on the environment but also on several organisms as aquatic species, plants, animals and humans (Ferk et al., 2007; Lavorgna et al., 2016; Mulder et al., 2018; Russo et al., 2018; Wu et al., 2011; Zhang et al., 2015). Moreover, some of QACs were considered endocrine disruptors and resulted in disturbances in the endocrine system of different organisms with subsequent alterations in reproduction and development (Melin et al., 2016; Sreevidya et al., 2018).

Due to these limitations and side effects of the conventional synthetic antimicrobial agents, the interest in biosourced antimicrobials as natural alternatives has increased remarkably. EOs are being favored as no particular resistance have been developed against them as they are composed of different active components, have multiple targets in bacterial cells and subsequently multiple modes of antimicrobial activity (Bakkali et al., 2008; García-Salinas et al., 2018; Kavanaugh and Ribbeck, 2012; Vidács et al., 2018). Additionally, as the chemical composition of each batch may be different, it would be difficult for microorganisms to develop a mechanism of resistance against EOs (Sieniawska et al., 2013). However, it should be pointed out that microbial strains could have different sensitivities to EOs (Marotta et al., 2016) and they might show as well a decreased susceptibility to some EOs due to the repeated exposure (Becerril et al., 2012). Therefore, further studies should explore the possible antimicrobial tolerance or resistance mechanisms that could be developed against EOs over time.

## 4 Combined essential oils and their active components with other treatments

### 4.1 Micro- and nano-encapsulated essential oils and their active components

Several strategies have been adopted to encounter the different limitations and improve the antimicrobial activity of free EOs including the development of new delivery systems as micro- and nano-capsules. The different encapsulation systems entrapping EOs were able to reduce or to eliminate microorganisms in different states as vegetative (Esmaeili and Asgari, 2015; Granata et al., 2018; Guarda et al., 2011; Jamil et al., 2016; Shetta et al., 2019), and biofilms (Giongo et al., 2016; Mechmechani et al., 2022; Rossi et al., 2017; Yammine et al., 2022). Moreover, encapsulated EOs reduced the viability of spores, inhibited molds growth as well as the formation of mycotoxins (Jiang et al., 2023; López-Meneses et al., 2018; Meng et al., 2022; Wang et al., 2018). Until present, and despite remaining a public health concern, no studies as to our knowledge have yet reported the antimicrobial activity of encapsulated EOs against VBNC cells.

The enhanced antimicrobial activity of the encapsulated EOs against the different types of microbial contaminations could be mainly linked to the reduced size of the particles and their increase in surface to volume ratio, which facilitates the diffusion of EOs into microbial cells (Cui et al., 2016; Granata et al., 2018). Also, the diffusion of EOs to cell membranes is facilitated by the encapsulation process as free EOs have low water solubility and thus a difficulty to interact with membranes whereas encapsulated EOs have an enhanced solubility (Moghimi et al., 2016). Encapsulation protects also EOs from degradation and volatilization which could be additionally related to a possible enhancement of their antimicrobial activity (da Silva Gündel et al., 2018).

#### 4.1.1 Against vegetative cells

Several studies attempted to reduce considerably vegetative bacterial cells using encapsulated EOs. The encapsulation of *Carum copticum* EO enhanced its antimicrobial activity against *S. epidermidis*, *S. aureus*, *B. cereus*, *E. coli*, *S.* Typhimurium and *P. vulgaris* (Esmaeili and Asgari, 2015). Granata et al. (2018) reported a bactericidal activity of encapsulated *Thymus capitata* and *Origanum vulgare* EOs against pathogenic bacteria, namely *S. aureus*, *E. coli*, *P. aeruginosa* and *L. monocytogenes*. The encapsulation of EOs active components carvacrol and thymol promoted the growth inhibition of a broad spectrum of microorganisms such as *S. aureus*, *E. coli* O157:H7, *L. innocua* and *S. cerevisiae* (Guarda et al., 2011). Nanoencapsulated cardamon, green tea and peppermint EOs showed similar trends by exhibiting excellent antimicrobial potencies against *E. coli* and *S. aureus* (Jamil et al., 2016; Shetta et al., 2019). Moreover, several studies investigated the susceptibility of Gram-negative and Gram-positive bacteria to encapsulated EOs. Gram-positive bacteria were found to be more susceptible to the antimicrobial activity of several encapsulated EOs as compared to the Gram-negative bacteria (Esmaeili and Asgari, 2015; Granata et al., 2018; Guarda et al., 2011). This could be due to the additional outer membrane containing lipopolysaccharides in Gram-negative bacteria that obstructs the penetration of EOs.

Moreover, most of literature studies showed an enhanced antimicrobial activity of EOs when encapsulated into different carrier systems with lower MIC values compared to their free forms. Exceptionally, some EOs or their active components showed equivalent MIC values in their free and encapsulated forms, like *Cymbopogon flexuosus*, peppermint and carvacrol against *S. aureus*, *E. coli* and *E. faecalis* (Table 3). Lower EOs concentrations used in the encapsulation process achieved equivalent or better antimicrobial activity against several microorganisms when compared to the free EOs (da Silva Gündel et al., 2018; Granata et al., 2018; Mechmechani et al., 2022; Moghimi et al., 2018; Shetta et al., 2019; Yammine et al., 2022). This will additionally reduce sensorial impacts on food products, economic costs, toxic side effects and any potential resistance that may be developed by microorganisms against EOs. Also, an improved long-term antimicrobial activity of encapsulated EOs was demonstrated. For example, the antimicrobial activity of eugenol and thymol was prolonged for 24 h against *E. coli* O157:H7 and *L. monocytogenes* when encapsulated (Chen et al., 2015). Cardamom EO antimicrobial activity was also prolonged for 7 days against *E. coli* and methicillin resistant *S. aureus* with encapsulation (Jamil et al., 2016). Free cinnamon, clove and thyme EOs presented an antimicrobial activity for maximum 2 days, while encapsulating them prolonged their activity to 8 days (Soliman et al., 2013). However, most of EOs encapsulation studies lack a direct application in food or other types of matrices with their subsequent sensory evaluations.

**Table 3:** Comparison of MIC values between free and encapsulated EOs and their active components against different types of microorganisms.

Microorganisms (in planktonic state)	EOs or active EO components	MIC approximate range (mg/ml)		Reference
		Free	Encapsulated	
**Gram-positive bacteria**				
*E. faecalis*	Carvacrol	0.62	0.62	Mechmechani et al. (2022)
*M. fortuitum*	*Cymbopogon flexuosus*	0.87	0.35	Rossi et al. (2017)
*M. massiliense*		3.50	0.35	
*M. abscessus*		3.50	0.35	
*S. aureus*	*Mentha piperita*	1.36	1.11	Shetta et al. (2019)
	*Camellia sinensis*	5.44	0.57	Shetta et al. (2019)
	*Origanum vulgare*	4	0.5	Granata et al. (2018)
	*Thymus capitatus*	2	0.25	Granata et al. (2018)
	*Cymbopogon flexuosus*	0.58	0.58	Rossi et al. (2017)
	Cardamon	4.4	0.27	Nahr et al. (2018)
*L. monocytogenes*	*Origanum vulgare*	2	1	Granata et al. (2018)
*L. delbrueckii*	D-limonene	>25	10	Donsì et al. (2011)
**Gram-negative bacteria**				
*S*. Enteritidis	Carvacrol	1.25	0.31	Yammine et al. (2022)
	Thymol	1.25	0.62	
*P. aeruginosa*	Carvacrol	5	1.25	Mechmechani et al. (2022)
	*Cymbopogon flexuosus*	-∗	>11.33	Rossi et al. (2017)
*E. coli*	*Thymus daenensis*	4.0	0.4	Moghimi et al. (2016)
	*Mentha piperita*	2.72	2.72	Shetta et al. (2019)
	*Camellia sinensis*	5.44	1.15	Shetta et al. (2019)
	*Origanum vulgare*	4	0.5	Granata et al. (2018)
	*Thymus capitatus*	2	0.25	Granata et al. (2018)
	Cardamon	4.4	0.27	Nahr et al. (2018)
	D- limonene	>25	10	Donsì et al., 2011
*A. baumannii*	*Thymus daenensis*	0.06–0.08	0.03–0.04	Moghimi et al. (2018)
**Fungi**				
*C. albicans*	*Cymbopogon flexuosus*	1.22	0.28	Rossi et al. (2017)
*C. grubii*		0.58	0.28	
*S. cerevisiae*	D- limonene	>25	10	Donsì et al., 2011

-∗No inhibitory activity detected at all tested concentrations.

#### 4.1.2 Against microbial spores

Encapsulated EOs showed prominent activity against microbial spores by inhibiting mainly spore germination or the spore-forming microorganisms. For example, the germination of both *Fusarium graminearum* and *Phomopsis* sp. were inhibited once exposed to encapsulated cinnamon and lemon EOs, respectively (Meng et al., 2022; Wu et al., 2019). Moreover, encapsulated clove EO suppressed the activity of *Penicillium digitatum* spores' germination as well as the elongation of the germ tube (Wang et al., 2018). The antimicrobial activity of encapsulated EOs was also tested against spore-forming bacteria and results have shown that encapsulation of Mānuka EO inactivated 4 logs of spore-forming *B. cereus* as compared to only 1 log reduction induced by the non-encapsulated EO (Liu et al., 2021). Moreover, *B. cereus* were reduced significantly in rice samples with up to 81.88% release of intracellular DNA, proteins and ATP after exposure to encapsulated curry plant EO (Cui et al., 2017). Despite presenting high risks, studies on the activity of encapsulated EOs against bacterial spores are limited compared to their antimicrobial activity against the other types of microbial contaminations.

#### 4.1.3 Against molds and mycotoxins

Encapsulated EOs exhibited also a remarkable activity against molds and their associated mycotoxins. Results showed that the encapsulation of several EOs together promoted their antifungal activity against *Aspergillus flavus* as well as their anti-aflatoxigenic potency by downregulating *ver-1* and *omt-A* genes function (Kumar et al., 2019). Other studies showed that encapsulation of cinnamon and hop EOs enhanced their antifungal activities against *Fusarium graminearum* by inhibiting mycelial growth, and by remarkably suppressing the production of the deoxynivalenol mycotoxin in rice samples (Jiang et al., 2023; Wu et al., 2019). The growth of *Aspergillus parasiticus* was inhibited after exposure to nanoencapsulated *Schinus molle* L. EO with a 59% reduction in Aflatoxin production (López-Meneses et al., 2018). In beef patties samples, yeasts and mold counts were reduced to up to 3.16 logs after exposure to encapsulated cinnamon EO (Ghaderi-Ghahfarokhi et al., 2017). Moreover, Aflatoxin B1 levels were effectively reduced in *Salvia hispanica* seeds once treated with nanoencapsulated *Zingiber zerumbet* EO (Deepika et al., 2021). Lemon EO nanoemulsions significantly inhibited mycelial growth of *Phomopsis* sp. preventing rot development and postharvest decay of fresh kiwi fruits. This inhibitory effect was ascribed to the enhanced activities of the intracellular antioxidant enzymes, to the accumulation of reactive oxygen species, and to cell apoptosis (Meng et al., 2022). Wang et al. (2018), and Hasheminejad et al. (2019) reported a superior ability of encapsulated clove EO to control *Penicillium digitatum* green molds developed on Navel oranges, as well as the mycelial growth of *Aspergillus niger* in pomegranate, respectively. The activity of encapsulated EOs on molds and their associated mycotoxins was related to several factors including the type and concentrations of EOs used as well as the food matrix components.

#### 4.1.4 Against biofilms

Increasing number of studies showed the promising antibiofilm activity of encapsulated EOs. They have reported damages to the biofilm matrix and even an eradication of biofilms. As reported by Yammine et al. (2022), nanoencapsulation of carvacrol and thymol inhibited totally *S*. Enteritidis biofilms after an exposure for 15 min at 2 MIC. While, free carvacrol and thymol at the same exposure time and concentration reduced up to 4.27 logs of biofilm cells. Similarly, nanoencapsulation of geranium EO inhibited significantly Candida spp. biofilm formation as compared to lower activity induced by free EO (Giongo et al., 2016). Mechmechani et al., 2022a, Mechmechani et al., 2022b demonstrated the higher antibiofilm activity of encapsulated carvacrol against *P. aeruginosa* and *E. faecalis* biofilms as compared to free EOs components. Nanoencapsulated *Cymbopogon flexuosus* showed a complete eradication of several strains of Mycobacteria biofilms (Rossi et al., 2017). Moreover, scanning electron microscopy images confirmed the morphological damages induced to bacterial cells recovered from biofilms after exposure to encapsulated EOs (Yammine et al., 2022). This proves that encapsulated EO could penetrate and disrupt the biofilms complex matrix and subsequently induce damages and death to bacterial cells.

### 4.2 Combining essential oils and their active components with other treatments

Strategies to control microbial contaminations are currently oriented toward multiple-hurdle technology such as the combination of EOs with other treatments (Calo et al., 2015; He et al., 2021; Mechmechani et al., 2022; Prakash et al., 2018). The main purpose of this combination is to observe a synergistic effect that provides a combined effect greater than the sum of individual effects. When combining EOs with other hurdles, synergism may be due to: i) the different modes of actions of the treatments applied and thus their different target sites, ii) to the interaction of one antimicrobial with cell membrane or cell wall leading to an increase uptake of other agents, or iii) to the inhibition of a series of steps in a biochemical pathway (Hyldgaard et al., 2012). Recent studies explored the combination of free and encapsulated EOs with other hurdles as heat, ultra-sound (US), high pressure processing (HPP), irradiation, pulsed electric field (PEF), vacuum packaging (VP), modified atmosphere packaging (MAP), antibiotics or other antimicrobial agents (Table 4). All the different combinations with EOs showed a significant synergistic effect except when coriander EO was combined with some antibiotics, an additive effect was observed and when oregano EO was combined with PEF, no synergism was noticed (Duarte et al., 2012; Clemente et al., 2020).

**Table 4:** Antimicrobial activity of EOs and their active components in combination with other hurdles.

Target microorganisms	EOs or active EO components (Concentration)	Combined treatment	Exposure time, Application	Antimicrobial activity		References
				EO or other treatment alone	Combined treatment	
Free						
*E. coli* O157:H7, *L. monocytogenes*	*Citrus sinensis* (0.2 μl), *Citrus lemon* (0.2 μl), *Citrus reticulata* (0.2 μl)	Mild heat (54 °C)	10 min, Lab culture	No inactivation	More than 5 log cycles inactivation	Espina et al. (2011)
*Cronobacter sakazakii* (Desiccated and non-desiccated)	Citral (0.3–0.9%)	Mild heat (25–55 °C)	2 h, Infant formula	N/A^a^	Complete inhibition	Shi et al. (2017)
Total viable counts*, Pseudomonas* spp., Lactic acid bacteria, *E. coli*, Total coliforms*,* *B. thermosphacta*, Yeasts and Molds	Thymol (0.4–0.8%) + Carvacrol (0.4–0.8%)	VP^b^	21 days, Marinated chicken and beef (Shawarma)	0.5–2.9 log reductions	0.8–3.1 log reductions	Karam et al. (2019), Karam et al. (2020)
Total mesophilic counts	*Cymbopogon citratus* (400 μl)	MAP^c^ (CO_2_ 100%)	21 days, Cabbage and radish sprouts	1.55–2.26 log reductions	Complete inactivation (<1 log) after 14 days	Hyun et al. (2015)
*Salmonella*	Thyme (0.3–0.9%)	VP, MAP (CO_2_ 50%)	15 days, Minced pork meat	Up to 2.9 log reductions in the first 3 days	1.69–4.05 log reductions in 15 days	Boskovic et al. (2017)
Total aerobic count, *B. cereus,* *C. perfringens*	*Origanum vulgare* (400–32000 ppm)	High pressure CO_2_ (100 bar/80 °C)	15 min-48 h, Paprika spice	Around 0.3 log reductions and no inactivation of spore population	Significant reductions and spores inactivation	Casas et al. (2016)
*Acinetobacter baumannii*	*Coriandrum sativum* L. (0–6 μl/ml)	Chloramphenicol (32–64 μl/ml)	16–20 h, Lab culture	MIC ranged between 1 and 4 μl/ml.	MIC of EO and antibiotic decreased by up to 62.5 times	Duarte et al. (2012)
*Acinetobacter baumannii*	*Myrtus communis* L. (0.03–1 MIC^d^)	Polymixin B, Ciprofloxacine (0.03–4 MIC)	24 h, Lab culture	MIC ranged between 0.25 and 4 μl/ml	MIC of antibiotics and EO reduced by up to 1/8 MIC with complete inhibition of microbial counts after 6 h	Aleksic et al. (2014)
*L. monocytogenes*, *E. coli* O157:H7	Oregano (0.01%), Lemongrass (0.01%)	Gamma irradiation (0.5 or 1 kGy)	14 days, Fresh cut cauliflower	1.16–3.29 log reductions	Undetectable up to 14 days at 0.5–1 kGy	Tawema et al. (2016)
Yeasts and molds				0.52–2.61 log reductions	Around 2–3 log reductions	
*Fusarium graminearum*	*Cananga odorata* (0–5 mg/g)	Gamma irradiation (0–10 kGy)	14 days, Maize	Complete inhibition at 3.9 mg/g	Significant reductions at 2.5 mg/g EO combined with 4 kGy irradiation	Kalagatur et al. (2018)
*A. niger*, *P. chrysogenum*	*Ocimum basilicum* (2%)	Irradiation (0–4 kGy)	14 days, Rice	0.42–1.18 log reductions	Up to 5 log reductions	Hossain et al. (2014)
*S.* Typhimurium	Clove (1.2 mg/ml)	Ultraviolet light-C	N/A, Lab culture	1.8–2.9 log reductions	6.8 log reductions	Silva-Espinoza et al. (2020)
*S.* Typhimurium	*Cinnamomum verum* (0–5%)	PEF^e^ (10–30 kV/cm)	60–3000 μs, Pasteurized skim milk	No bactericidal effect	Up to 1.97 log reductions	Pina-Pérez et al. (2012)
*Campylobacter jejuni*	Oregano (1/4 and 1/2 MIC = 15.625 and 31.25 ppm)	PEF (1–20 kV/cm)	20 μs, Liquids and raw chicken	1.64–3.32 log reductions with varied PEF treatments	EO applied following PEF induced further inactivation of 1.2 log cycles	Clemente et al. (2020)
*L. monocytogenes*	*Thymus vulgaris* L. (<0.06–0.50%)	HPP (200–300 MPa)	15 min, Fresh cheese	Around 3.5 log reductions	More than 5 log reductions	Bleoancă et al. (2016)
*L. monocytogene,* *L. innocua*	*Mentha piperita* (0.05 and 0.1 ml/100 ml)	HPP (600 MPa)	300 s, Yogurt drink	More than 5 log reductions	Additional 1 log reduction and reduced HPP treatment	Evrendilek and Balasubramaniam (2011)
*S.* Typhimurium, *L. monocytogenes*	*Cinnamomum zeylanicum* (0.625 μL/mL)	US (24 kHz, 400 W)	0–6 days, Milk	0.7–3.0 log reductions	2.7–4.5 log reductions	Mortazavi and Aliakbarlu (2019)
*S.* Typhimurium, *S. aureus*	*Zataria multiflora* Boiss. (15–30 μl/100 ml)	Nisin (0–0.5 μg/ml at 8 and 25 °C)	21 days, Barley soup	Complete inhibition in 2–12 days	Complete inhibition	Moosavy et al. (2008)
*L. monocytogenes*	*Origanum vulgare* L., *Thymus vulgaris* L., *Rosmarinus officinalis* L. (50–300 ppm)	Lactic acid (50 ppm)	24 h, Lab culture	32.0–83.48 % inhibition	60.33–99.08% inhibition	Dimitrijevic et al. (2007)
*S. epidermidis*	Thymol (0.06–16 g/l), Tea tree (0.25–64 g/l), *Eucalyptus* (0.25–64 g/l)	Chlorhexidine digluconate (0.125–16 mg/l)	24 h, Lab culture	MIC: 0.5–16 g/l	Reduced MIC to 0.5–1 g/l	Karpanen et al. (2008)
*S. epidermidis* (Biofilms)				MIC: 0.5–64 g/l	Reduced MIC to 0.25–16 g/l	
*Salmonella enterica* serotype Newport	*Origanum vulgare*, Carvacrol (0.1–0.5%)	Ozonized water (0.01–0.1 mg O_3_/L)	60–120 min, Iceberg lettuce	1.76–2.09 log reductions	Complete inhibition	Dev Kumar and Ravishankar (2019)
**Nano-encapsulated**						
*E. coli* O157:H7 Sakai*,* *L. monocytogenes* EGD-e	*Thymbra capitate* nanoparticles (0.1 μl)	Heat (53 °C)	12 min, Lab culture	Up to 0.5 log reductions	Up to 5 log reductions	Merino et al. (2019)
*A. niger*, *A. flavus*, *A.parasiticus*, *P. chrysogenum*	*Thymus vulgaris* + *Origanum compactum* nanocrystals (0.13 and 0.19%)	Irradiation (750 kGy)	8 weeks, Rice	1.19–2.87 log reductions	3.7–4.93 log reductions	Hossain et al. (2019)
*E. coli* O157:H7	Thyme nanoemulsions (0.375 mg/ml)	US^f^ (127–255 W/cm^2^)	3–9 min, Lab culture	3.28–4.13 log reductions	5.14–7.42 log reductions	Guo et al. (2020)
*S. enterica*	Oregano and thyme nanoemulsions (0.025%)	US (continuous and pulsed 200 W)	5–25 min, Lettuce leaves	Up to 2.23 log reductions	Complete inactivation	Millan-Sango et al. (2016)

a N/A: Not Applicable.

b VP: Vacuum Packaging.

c MAP: Modified Atmosphere Packaging.

d MIC: Minimum Inhibitory Concentration.

e PEF: Pulsed Electric Field.

f US: Ultrasound.

When EOs were combined with mild heating (53–55 °C), synergism was reported on the reduction and/or elimination of *E. coli* O157:H7, *L. monocytogenes* and *C. sakazakii* bacterial populations in lab cultures and in infant formulas (Espina et al., 2011; Merino et al., 2019; Shi et al., 2017).

Another promising intervention involves the combination of EOs with VP and MAP, which has been assessed for the packaging of different food products. Combination of EOs with VP and/or MAP showed a synergistic effect with an enhanced reduction of spoilage and/or pathogenic bacteria in marinated chicken samples (Karam et al., 2019), in paprika spices (Casas et al., 2016), in marinated beef (Karam et al., 2020), and in minced pork meat packages (Boskovic et al., 2017). Populations of total mesophilic counts in cabbage and radish sprouts were completely inactivated when lemongrass EO was combined with MAP (Hyun et al., 2015).

MIC of different EOs were reduced when combining them with conventional antibiotics such as Chloramphenicol, Ciprofloxacin, Gentamicin, Tetracycline and Polymixin B, in lab cultures (Aleksic et al., 2014; Duarte et al., 2012). Moreover, a synergistic antibiofilm activity of several EOs was reported when combined with common antibiotics as Norfloxacin, Gentamicin, Oxacillin, Tetracycline and Ampicillin against different strains of Gram-positive and Gram-negative biofilms (El Baaboua et al., 2015; Rosato et al., 2020). This combination reduced the amounts of both EOs and antibiotics used to control microbial contamination and biofilms. However, another study reported an additive effect against *Acinetobacter baumannii* when coriander EO was combined with Cefoperazone and Piperacillin antibiotics (Duarte et al., 2012).

An enhanced antimicrobial activity of EOs was also reported when combined with irradiation against *L. monocytogenes*, *E. coli* O157:H7, yeasts and molds, *A. niger*, *Aspergillus flavus*, *Aspergillus parasiticus* and *Penicillium chrysogenum* in cauliflower and rice (Hossain et al. 2014, 2019; Tawema et al., 2016). Additionally, a complete inhibition of *S.* Typhimurium biofilms, *Fusarium graminearum* mycotoxins and an absence of intracellular ATP within *L. monocytogenes* and *E. coli* O157:H7 populations were observed in lab cultures as well as in maize samples (Caillet et al., 2005; Caillet and Lacroix, 2006; Kalagatur et al., 2018; Silva-Espinoza et al., 2020).

Similarly, when EOs were combined with PEF, a synergistic effect was observed against *S.* Typhimurium and *E. coli* O157:H7 populations in pasteurized skim milk and in different juices, respectively (Ait-Ouazzou et al., 2013; Pina-Pérez et al., 2012). However, when oregano was combined with PEF, the antimicrobial activity of oregano did not improve against *C. jejuni* in liquids and on raw chicken, except when following a sequential combination approach and applying EO after the PEF treatment, a significant increase in inactivation levels was reported (Clemente et al., 2020).

When applying HPP in combination with EOs, an enhanced antimicrobial activity was observed against *L. monocytogenes* and *L. innocua* in fresh cheese and yogurt drink, respectively (Bleoancă et al., 2016; Evrendilek and Balasubramaniam, 2011). This combination resulted in a reduction of pressure severity and impacts on food products.

The application of US treatment in combination with EOs also resulted in enhanced antimicrobial activity against *E. coli* 0157:H7 and a reduction of *S. enterica* populations below detection limits in lettuce leaves (Guo et al., 2020; Millan-Sango et al., 2016). Also, in low and high fat milk samples, cinnamon EO combined with US treatment induced further significant reductions in *S.* Typhimurium and *L. monocytogenes* populations compared to individual treatments (Mortazavi and Aliakbarlu, 2019).

Additionally, when combined with other antimicrobial agents (such as nisin, lactic acid, diglycerol monolaurate or chlorhexidine digluconate), EOs showed remarkable synergistic effects against *L. monocytogenes*, *B. subtilis* and, *S. epidermidis* (Dimitrijevic et al., 2007; Ettayebi et al., 2000; Karpanen et al., 2008; Yamazaki et al., 2004; Yoon et al., 2011).

The combination of an optimized mixture of EOs with other hurdles showing synergistic effect may reduce the concentrations required to yield the same antimicrobial activity when compared to EOs used alone. This may result in lower economic costs and lower sensorial impacts on food products, while maintaining their microbiological safety (Hyldgaard et al., 2012). Also, combining EOs with conventional antibiotics may reduce the latter used concentrations and thus decrease the probability of developing resistance towards them and minimize their side effects.

## 5 Conclusions

In this review, the advances and major trends in the use of EOs as potent tools to fight microbial contaminations were addressed. Additionally, the functional role and mechanisms of action of the different active EO components were discussed. Among the different active EO components, phenolic compounds were found to exhibit the greatest antimicrobial activity by acting principally on the disruption and increase in permeability of cell membranes, inducing a leakage of vital intracellular contents and eventually leading to microorganisms' inactivation and death. In most studies, the antimicrobial activity of active compounds was more pronounced than that of whole EOs, thus minimizing the amounts of EOs being used while achieving more reproducibility and standardization. EOs and their active components were effectively used in their free, micro- and nano-encapsulated forms and combined with other hurdles for antimicrobial purposes. Generally, encapsulation of EOs and their combination with other hurdles provided an enhanced antimicrobial activity when compared to free EO applications, with lower amounts being used. This could be promising technologies to control the different types of microbial contaminations and achieve microbiological safety. Further research studies should explore: i) the antimicrobial mode of action of EOs combined with other hurdles as one strategy could promote or modify the action of the other; ii) the combination of encapsulated EOs with other hurdles to determine their potential antimicrobial efficiencies as it is expected to have a synergistic or additive effect; and iii) the impact of the strong odor of EOs on the sensorial properties of food products. Additional studies should also assess the activities of EOs encapsulated or combined with other hurdles against microorganisms in different states as biofilms, viable but not culturable cells, spores and molds. Moreover, robust national and international frameworks and regulations should be set towards the use of EOs and their active components before being applied in diverse applications and fields.

## Declarations

### Author contribution statement

All authors listed have significantly contributed to the development and the writing of this article.

### Funding statement

This work was supported by the Partenariat Hubert Curien (PHC)- Cèdre program [42281SD] and the open access funding was provided by the Qatar National Library.

### Data availability statement

No data was used for the research described in the article.

### Declaration of interest’s statement

The authors declare no competing interests.

### Additional information:

No additional information is available for this paper.

## References

[bib1] Abdallah M., Benoliel C., Drider D., Dhulster P., Chihib N.E. (2014). Biofilm Formation and persistence on abiotic surfaces in the context of food and medical environments. Arch. Microbiol..

[bib2] Adukwu E.C., Allen S.C.H., Phillips C.A. (2012). The anti-biofilm activity of lemongrass (*Cymbopogon flexuosus*) and Grapefruit (*Citrus Paradisi*) essential oils against five strains of *Staphylococcus aureus*. J. Appl. Microbiol..

[bib3] Afonso T.B., Simões L.C., Lima N. (2020). Effect of quorum sensing and quenching molecules on inter-kingdom biofilm formation by Penicillium expansum and bacteria. Biofouling.

[bib4] Aiemsaard J., Aiumlamai S., Aromdee C., Taweechaisupapong S., Khunkitti W. (2011). The effect of lemongrass oil and its major components on clinical isolate mastitis pathogens and their mechanisms of action on *Staphylococcus aureus* DMST 4745. Res. Vet. Sci..

[bib5] Ait-Ouazzou A., Espina L., García-Gonzalo D., Pagán R. (2013). Synergistic combination of physical treatments and carvacrol for *Escherichia coli* O157:H7 inactivation in apple, mango, orange, and tomato juices. Food Control.

[bib6] Aleksic Sabo V., Knezevic P. (2019). Antimicrobial activity of Eucalyptus camaldulensis Dehn. Plant extracts and essential oils: a review. Ind. Crop. Prod..

[bib7] Aleksic V., Mimica-Dukic N., Simin N., Nedeljkovic N., Knezevic P. (2014). Synergistic effect of myrtus communis L. Essential oils and conventional antibiotics against multi-drug resistant *Acinetobacter baumannii* wound isolates. Phytomedicine.

[bib8] Ali S., Khan A., Ahmed I., Musaddiq M., Ahmed K., Polasa H., Venkateswar Rao L., Habibullah C., Sechi L., Ahmed N. (2005). Antimicrobial activities of eugenol and cinnamaldehyde against the human gastric pathogen *Helicobacter pylori*. Ann. Clin. Microbiol. Antimicrob..

[bib9] Al-Maqtari Q.A., Rehman A., Mahdi A.A., Al-Ansi W., Wei M., Yanyu Z., Phyo H.M., Galeboe O., Yao W. (2021). Application of essential oils as preservatives in food systems: challenges and future prospectives – a review. Phytochemistry Rev..

[bib11] Almatroudi A., Hu H., Deva A., Gosbell I.B., Jacombs A., Jensen S.O., Whiteley G., Glasbey T., Vickery K. (2015). A new dry-surface biofilm model: an essential tool for efficacy testing of hospital surface decontamination procedures. J. Microbiol. Methods.

[bib12] Almatroudi A., Shamaila T., Honghua H., Durdana C. (2018). *Staphylococcus aureus* dry-surface biofilms are more resistant to heat treatment than traditional hydrated biofilms. J. Hosp. Infect..

[bib13] Almeida L.D.F.D.D., Paula J.F.D., de Almeida R.V.D., Williams D.W., Hebling J., Cavalcanti Y.W. (2016). Efficacy of citronella and cinnamon essential oils on *Candida albicans* biofilms. Acta Odontol. Scand..

[bib14] Amalaradjou M.A.R., Venkitanarayanan K. (2011). Effect of trans-cinnamaldehyde on inhibition and inactivation of *Cronobacter sakazakii* biofilm on abiotic surfaces. J. Food Protect..

[bib15] Amaral V.C.S., Santos P.R., da Silva A.F., dos Santos A.R., Machinski M., Graton Mikcha J.M. (2015). Effect of carvacrol and thymol on *Salmonella* spp. Biofilms on polypropylene. Int. J. Food Sci. Technol..

[bib16] Bakkali F., Averbeck S., Averbeck D., Idaomar M. (2008). Biological effects of essential oils – a review. Food Chem. Toxicol..

[bib17] Barriuso J., Hogan D.A., Keshavarz T., Martínez M.J. (2018). Role of quorum sensing and chemical communication in fungal biotechnology and pathogenesis. FEMS Microbiol. Rev..

[bib18] Bazargani M.M., Rohloff J. (2016). Antibiofilm activity of essential oils and plant extracts against *Staphylococcus aureus* and *Escherichia coli* biofilms. Food Control.

[bib19] Becerril R., Nerín C., Gómez-Lus R. (2012). Evaluation of bacterial resistance to essential oils and antibiotics after exposure to oregano and cinnamon essential oils. Foodborne Pathog. Dis..

[bib20] Belessi C.E.A., Gounadaki A.S., Psomas A.N., Skandamis P.N. (2011). Efficiency of different sanitation methods on *Listeria monocytogenes* biofilms formed under various environmental conditions. Int. J. Food Microbiol..

[bib21] Ben Hsouna A., Ben Halima N., Smaoui S., Hamdi N. (2017). Citrus lemon essential oil: chemical composition, antioxidant and antimicrobial activities with its preservative effect against *Listeria monocytogenes* inoculated in minced beef meat. Lipids Health Dis..

[bib22] Bhargava K., Conti D.S., da Rocha S.R.P., Zhang Y. (2015). Application of an oregano oil nanoemulsion to the control of foodborne bacteria on fresh lettuce. Food Microbiol..

[bib23] Bhavaniramya S., Vishnupriya S., Al-Aboody M.S., Vijayakumar R., Baskaran D. (2019). Role of essential oils in food safety: antimicrobial and antioxidant applications. Grain Oil Sci. Technol..

[bib24] Bleoancă I., Saje K., Mihalcea L., Oniciuc E.A., Smole-Mozina S., Nicolau A.I., Borda D. (2016). Contribution of high pressure and thyme extract to control *Listeria monocytogenes* in fresh cheese - a hurdle approach. Innovat. Food Sci. Emerg. Technol..

[bib25] Boehnke K.F., Eaton K.A., Fontaine C., Brewster R., Wu J., Eisenberg J.N.S., Valdivieso M., Baker L.H., Xi C. (2017). Reduced infectivity of waterborne viable but nonculturable *Helicobacter pylori* strain SS1 in mice. Helicobacter.

[bib26] Boskovic M., Djordjevic J., Ivanovic J., Janjic J., Zdravkovic N., Glisic M., Glamoclija N., Baltic B., Djordjevic V., Baltic M. (2017). Inhibition of *Salmonella* by thyme essential oil and its effect on microbiological and sensory properties of minced pork meat packaged under vacuum and modified atmosphere. Int. J. Food Microbiol..

[bib27] Bouhdid S., Abrini J., Zhiri A., Espuny M.J., Manresa A. (2009). Investigation of functional and morphological changes in *Pseudomonas aeruginosa* and *Staphylococcus aureus* cells induced by *Origanum compactum* essential oil. J. Appl. Microbiol..

[bib28] Bouyahya A., Abrini J., Dakka N., Bakri Y. (2019). Essential oils of *Origanum Compactum* increase membrane permeability, disturb cell membrane integrity, and suppress quorum-sensing phenotype in bacteria. J. Pharm. Anal..

[bib29] Brackman G., Defoirdt T., Miyamoto C., Bossier P., Van Calenbergh S., Nelis H., Coenye T. (2008). Cinnamaldehyde and cinnamaldehyde derivatives reduce virulence in Vibrio spp. by decreasing the DNA-binding activity of the quorum sensing response regulator LuxR. BMC Microbiol..

[bib30] Braga P.C., Culici M., Alfieri M., Dal Sasso M. (2008). Thymol inhibits *Candida albicans* biofilm formation and mature biofilm. Int. J. Antimicrob. Agents.

[bib31] Brenes A., Roura E. (2010). Essential oils in poultry nutrition: main effects and modes of action. Anim. Feed Sci. Technol..

[bib32] Bridier A., Briandet R., Thomas V., Dubois-Brissonnet F. (2011). Resistance of bacterial biofilms to disinfectants: a review. Biofouling.

[bib33] Burgess S.A., Lindsay D., Flint S.H. (2010). Thermophilic Bacilli and their importance in dairy processing. Int. J. Food Microbiol..

[bib34] Burt S.A., van der Zee R., Koets A.P., de Graaff A.M., van Knapen F., Gaastra W., Haagsman H.P., Veldhuizen E.J.A. (2007). Carvacrol induces heat shock protein 60 and inhibits synthesis of flagellin in *Escherichia coli* O157:H7. Appl. Environ. Microbiol..

[bib35] Burt S. (2004). Essential oils: their antibacterial properties and potential applications in foods—a review. Int. J. Food Microbiol..

[bib36] Caillet S., Lacroix M. (2006). Effect of gamma radiation and oregano essential oil on murein and ATP concentration of *Listeria monocytogenes*. J. Food Protect..

[bib37] Caillet S., Shareck F., Lacroix M. (2005). Effect of gamma radiation and oregano essential oil on murein and ATP concentration of *Escherichia coli* O157:H7. J. Food Protect..

[bib38] Çakmak H., Özselek Y., Turan O.Y., Firatligil E., Karbancioglu-Güler F. (2020). Whey protein isolate edible films incorporated with essential oils: antimicrobial activity and barrier properties. Polym. Degrad. Stabil..

[bib39] Calo J.R., Crandall P.G., O’Bryan C.A., Ricke S.C. (2015). Essential oils as antimicrobials in food systems – a review. Food Control.

[bib40] Campana R., Casettari L., Fagioli L., Cespi M., Bonacucina G., Baffone W. (2017). Activity of essential oil-based microemulsions against *Staphylococcus aureus* biofilms developed on stainless steel surface in different culture media and growth conditions. Int. J. Food Microbiol..

[bib41] Casas J., Tello J., Gatto F., Calvo L. (2016). Microbial inactivation of paprika using oregano essential oil combined with high-pressure CO2. J. Supercrit. Fluids.

[bib42] Casasola-Rodríguez B., Ruiz-Palacios G.M., Pilar R.C., Losano L., Ignacio M.R., Orta de Velásquez M.T. (2018). Detection of VBNC *Vibrio cholera* by RT-real time PCR based on differential gene expression analysis. FEMS Microbiol. Lett..

[bib43] Cava R., Nowak E., Taboada A., Marin-Iniesta F. (2007). Antimicrobial activity of clove and cinnamon essential oils against *Listeria monocytogenes* in pasteurized milk. J. Food Protect..

[bib44] Chang C.W., Lin M.H. (2017). Optimization of PMA-qPCR for *Staphylococcus aureus* and determination of viable bacteria in indoor air. Indoor Air.

[bib45] Chang S.T., Chen P.F., Chang S.C. (2001). Antibacterial activity of leaf essential oils and their constituents from Cinnamomum Osmophloeum. J. Ethnopharmacol..

[bib46] Charlebois A., Jacques M., Boulianne M., Archambault M. (2017). Tolerance of *Clostridium perfringens* biofilms to disinfectants commonly used in the food industry. Food Microbiol..

[bib47] Chavant P., Gaillard-Martinie B., Hébraud M. (2004). Antimicrobial effects of sanitizers against planktonic and sessile *Listeria monocytogenes* cells according to the growth phase. FEMS Microbiol. Lett..

[bib48] Chen H., Zhang Y., Zhong Q. (2015). Physical and antimicrobial properties of spray-dried Zein–Casein nanocapsules with Co-encapsulated eugenol and thymol. J. Food Eng..

[bib49] Chouhan S., Sharma K., Guleria S. (2017). Antimicrobial activity of some essential oils—present status and future perspectives. Medicines.

[bib50] Clemente I., Condón-Abanto S., Pedrós-Garrido S., Whyte P., Lyng J.G. (2020). Efficacy of pulsed electric fields and antimicrobial compounds used alone and in combination for the inactivation of *Campylobacter jejuni* in liquids and raw chicken. Food Control.

[bib52] Cui H., Li W., Li C., Vittayapadung S., Lin L. (2016). Liposome containing cinnamon oil with antibacterial activity against methicillin-resistant *Staphylococcus aureus* biofilm. Biofouling.

[bib53] Cui H., Li W., Lin L. (2017). Antibacterial activity of liposome containing curry plant essential oil against *Bacillus cereus* in rice. J. Food Saf..

[bib54] Cui H., Zhang X., Zhou H., Zhao C., Lin L. (2015). Antimicrobial activity and mechanisms of Salvia sclarea essential oil. Bot. Stud..

[bib55] da Silva Gündel S., de Souza M.E., Quatrin P.M., Klein B., Wagner R., Gündel A., de Almeida Vaucher R., Santos R.C.V., Ferreira Ourique A. (2018). Nanoemulsions containing cymbopogon flexuosus essential oil: development, characterization, stability study and evaluation of antimicrobial and antibiofilm activities. Microb. Pathog..

[bib56] Deepika S.A., Chaudhari A.K., Das S., Dubey N.K. (2021). Zingiber zerumbet L. Essential oil based chitosan nanoemulsion as an efficient green preservative against fungi and Aflatoxin B1 contamination. J. Food Sci..

[bib57] de Medeiros Barbosa I., da Costa Medeiros J.A., de Oliveira K.Á.R., Gomes-Neto N.J., Tavares J.F., Magnani M., de Souza E.L. (2016). Efficacy of the combined application of oregano and rosemary essential oils for the control of *Escherichia coli, Listeria monocytogenes* and *Salmonella enteritidis* in leafy vegetables. Food Control.

[bib58] de Oliveira M.M.M., Brugnera D.F., do Nascimento J.A., Batista N.N., Hilsdorf Piccoli R. (2012). Cinnamon essential oil and cinnamaldehyde in the control of bacterial biofilms formed on stainless steel surfaces. Eur. Food Res. Technol..

[bib59] de Oliveira M.M.M., Brugnera D.F., Cardoso M.D.G., Alves E., Piccoli R.H. (2010). Disinfectant action of cymbopogon sp. Essential oils in different phases of biofilm formation by *Listeria monocytogenes* on stainless steel surface. Food Control.

[bib60] Desai M.A., Soni K.A., Nannapaneni R., Schilling M.W., Silva J.L. (2012). Reduction of *Listeria monocytogenes* biofilms on stainless steel and polystyrene surfaces by essential oils. J. Food Protect..

[bib61] Dev Kumar G., Ravishankar S. (2019). Ozonized water with plant antimicrobials: an effective method to inactivate *Salmonella enterica* on iceberg lettuce in the produce wash water. Environ. Res..

[bib62] Dhifi W., Bellili S., Jazi S., Bahloul N., Mnif W. (2016). Essential oils’ chemical characterization and investigation of some biological activities: a critical review. Medicines.

[bib63] Dima C., Dima S. (2015). Essential oils in foods: extraction, stabilization, and toxicity. Curr. Opin. Food Sci..

[bib64] Dimitrijevic S.I., Mihajlovski K.R., Antonovic D.G., Milanovic-Stevanovic M.R., Mijin D.Z. (2007). A study of the synergistic antilisterial Effects of a sub-lethal dose of lactic acid and essential oils from Thymus vulgaris L., Rosmarinus Officinalis L. and Origanum vulgare L. Food Chem..

[bib65] Doležalová E., Prukner V., Lukeš P., Šimek M. (2016). Stress response of Escherichia coli induced by surface streamer discharge in humid air. J. Phys. Appl. Phys..

[bib66] Domadia P., Swarup S., Bhunia A., Sivaraman J., Dasgupta D. (2007). Inhibition of bacterial cell division protein FtsZ by cinnamaldehyde. Biochem. Pharmacol..

[bib67] Dong K., Pan H., Yang D., Rao L., Zhao L., Wang Y., Liao X. (2020). Induction, detection, formation and resuscitation of viable but non-culturable state microorganisms. Compr. Rev. Food Sci. Food Saf..

[bib68] Donlan R.M. (2002). Biofilms: microbial life on surfaces. Emerg. Infect. Dis..

[bib69] Donsì F., Annunziata M., Sessa M., Ferrari G. (2011). Nanoencapsulation of essential oils to enhance their antimicrobial activity in foods. LWT - Food Sci. Technol. (Lebensmittel-Wissenschaft -Technol.).

[bib70] dos Santos Rodrigues J.B., de Souza N.T., Scarano J.O.A., de Sousa J.M., Lira M.C., de Figueiredo R.C.B.Q., de Souza E.L., Magnani M. (2018). Efficacy of using oregano essential oil and carvacrol to remove young and mature *Staphylococcus aureus* biofilms on food-contact surfaces of stainless steel. LWT.

[bib71] dos Santos Rodrigues J.B., de Carvalho R.J., de Souza N.T., de Sousa Oliveira K., Franco O.L., Schaffner D., de Souza E.L., Magnani M. (2017). Effects of oregano essential oil and carvacrol on biofilms of *Staphylococcus aureus* from food-contact surfaces. Food Control.

[bib72] Du W., Zhou M., Liu Z., Chen Y., Li R. (2018). Inhibition effects of low concentrations of epigallocatechin gallate on the biofilm formation and hemolytic activity of *Listeria monocytogenes*. Food Control.

[bib73] Duarte A., Ferreira S., Silva F., Domingues F.C. (2012). Synergistic activity of coriander oil and conventional antibiotics against *Acinetobacter baumannii*. Phytomedicine.

[bib74] Ed-Dra A., Nalbone L., Filali F.R., Trabelsi N., El Majdoub Y.O., Bouchrif B. (2021). Comprehensive evaluation on the use of Thymus vulgaris essential oil as natural additive against different serotypes of *Salmonella enterica*. Sustainability.

[bib75] EFSA and ECDC (European Food Safety Authority and European Center for Disease Prevention and Control) (2018). The European summary report on trends and sources of Zoonoses, Zoonotic agents and food-borne outbreaks in 2017. EFSA J..

[bib76] El Abed S., Ibnsouda K.S., Latrache H., Zineb G., Mouradi H., Remmal A. (2011). Carvacrol and thymol components inhibiting *Pseudomonas aeruginosa* adherence and biofilm formation. Afr. J. Microbiol. Res..

[bib77] El Asbahani A., Miladi K., Badri W., Sala M., Aït Addi E.H., Casabianca H., El Mousadik A. (2015). Essential oils: from extraction to encapsulation. Int. J. Pharm..

[bib78] El Baaboua A., Maadoudi M., Bouyahya A., Belmehdi O., Kounnoun A., Cheyadmi S., Sanae O., Senhaji N.S., Abrini J. (2015). Evaluation of the combined effect of antibiotics and essential oils against Campylobacter multidrug resistant strains and their biofilm formation. South Afr. J. Bot..

[bib79] El Darier S.M., El-Ahwany A., Elkenany E.T., Abdeldaim A.A. (2018). An in vitro study on antimicrobial and anticancer potentiality of thyme and clove oils. Rendiconti Lincei. Sci. Fis. Nat..

[bib80] El Moussaoui N., Sanchez G., Khay E.O., Idaomar M., Ibn Mansour A., Abrini J., Aznar R. (2013). Antibacterial and antiviral activities of essential oils of northern Moroccan plants. Br. Biotechnol. J..

[bib81] El Sayed Zaki M., Bastawy S., Montasser K. (2019). Molecular study of resistance of *Staphylococcus aureus* to antiseptic quaternary ammonium compounds. J. Glob. Antimicrob. Resist..

[bib82] Esbelin J., Santos T., Hébraud M. (2018). Desiccation: an environmental and food industry stress that bacteria commonly face. Food Microbiol..

[bib83] Esmaeili A., Asgari A. (2015). In vitro release and biological activities of Carum copticum essential oil (CEO) loaded chitosan nanoparticles. Int. J. Biol. Macromol..

[bib84] Espina L., Somolinos M., Lorán S., Conchello P., García D., Pagán R. (2011). Chemical composition of commercial Citrus fruit essential oils and evaluation of their antimicrobial activity acting alone or in combined processes. Food Control.

[bib85] Ettayebi K., El Yamani J., Rossi-Hassani B.D. (2000). Synergistic effects of nisin and thymol on antimicrobial activities in *Listeria monocytogenes* and *Bacillus subtilis*. FEMS Microbiol. Lett..

[bib86] Evrendilek G.A., Balasubramaniam V.M. (2011). Inactivation of *Listeria monocytogenes* and *Listeria innocua* in yogurt drink applying combination of high pressure processing and mint essential oils. Food Control.

[bib87] Fakruddin M.D., Bin Mannan K.S., Andrews S. (2013). Viable but nonculturable bacteria: food safety and public health perspective. ISRN Microbiol..

[bib88] Falcó I., Verdeguer M., Aznar R., Sánchez G., Randazzo W. (2019). Sanitizing food contact surfaces by the use of essential oils. Innovat. Food Sci. Emerg. Technol..

[bib89] Ferk F., Misik M., Hoelzl C., Uhl M., Fuerhacker M., Grillitsch B., Parzefall W. (2007). Benzalkonium chloride (BAC) and dimethyldioctadecyl-ammonium bromide (DDAB), two common quaternary ammonium compounds, cause genotoxic effects in mammalian and plant cells at environmentally relevant concentrations. Mutagenesis.

[bib90] García-Salinas S., Elizondo-Castillo H., Arruebo M., Mendoza G., Irusta S. (2018). Evaluation of the antimicrobial activity and cytotoxicity of different components of natural origin present in essential oils. Molecules.

[bib91] Ghaderi-Ghahfarokhi M., Barzegar M., Sahari M.A., Ahmadi Gavlighi H., Gardini F. (2017). Chitosan cinnamon essential oil nano-formulations: application as a novel additive for controlled release and shelf life extension of beef patties. Int. J. Biol. Macromol..

[bib92] Gill A.O., Holley R.A. (2006). Disruption of *Escherichia coli, Listeria monocytogenes* and *Lactobacillus sakei* cellular membranes by plant oil aromatics. Int. J. Food Microbiol..

[bib93] Giongo J.L., de Almeida Vaucher R., Fausto V.P., Quatrin P.M., Lopes L.Q.S., Santos R.C.V., Gündel A., Gomes P., Steppe M. (2016). Anti- Candida activity assessment of Pelargonium graveolens oil free and nanoemulsion in biofilm formation in hospital medical supplies. Microb. Pathog..

[bib94] Granata G., Stracquadanio S., Leonardi M., Napoli E., Consoli G.M.L., Cafiso V., Stefani S., Geraci C. (2018). Essential oils encapsulated in polymer-based nanocapsules as potential candidates for application in food preservation. Food Chem..

[bib95] Guarda A., Rubilar J.F., Miltz J., Galotto M.J. (2011). The antimicrobial activity of microencapsulated thymol and carvacrol. Int. J. Food Microbiol..

[bib96] Guo J., He Z., Ma C., Li W., Wang J., Lin F., Liu X., Ling L. (2022). Evaluation of cold plasma for decontamination of molds and mycotoxins in rice grain. Food Chem..

[bib97] Guo M., Zhang L., He Q., Arabi S.A., Zhao H., Chen W., Ye X., Liu D. (2020). Synergistic antibacterial effects of Ultrasound and thyme essential oils nanoemulsion against *Escherichia coli* O157:H7. Ultrason. Sonochem..

[bib98] Gutierrez J., Barry-Ryan C., Bourke P. (2008). The antimicrobial efficacy of plant essential oil combinations and interactions with food ingredients. Int. J. Food Microbiol..

[bib99] Gutiérrez-del-Río I., Fernández J., Lombó F. (2018). Plant nutraceuticals as antimicrobial agents in food preservation: terpenoids, polyphenols and thiols. Int. J. Antimicrob. Agents.

[bib100] Han D., Hung Y.C., Wang L. (2018). Evaluation of the antimicrobial efficacy of neutral electrolyzed water on pork products and the formation of viable but nonculturable (VBNC) pathogens. Food Microbiol..

[bib101] Hasheminejad N., Khodaiyan F., Safari M. (2019). Improving the antifungal activity of clove essential oil encapsulated by Chitosan nanoparticles. Food Chem..

[bib102] Hayrapetyan H., Abee T., Groot M.N. (2016). Sporulation dynamics and spore heat resistance in wet and dry biofilms of *Bacillus cereus*. Food Control.

[bib103] He Q., Guo M., Jin T.Z., Arabi S.A., Liu D. (2021). Ultrasound improves the decontamination effect of thyme essential oil nanoemulsions against Escherichia coli O157:H7 on cherry tomatoes. Int. J. Food Microbiol..

[bib104] Hemaiswarya S., Soudaminikkutty R., Narasumani M.L., Doble M. (2011). Phenylpropanoids inhibit protofilament formation of *Escherichia coli* cell division protein FtsZ. J. Med. Microbiol..

[bib105] Highmore C.J., Warner J.C., Rothwell S.D., Wilks S.A., Keevil C.W. (2018). Viable-but-Nonculturable *Listeria monocytogenes* and *Salmonella enterica* serovar *thompson* induced by chlorine stress remain infectious. mBio.

[bib106] Hossain F., Follett P., Salmieri S., Vu K.D., Fraschini C., Lacroix M. (2019). Antifungal activities of combined treatments of irradiation and essential oils (EOs) encapsulated chitosan nanocomposite films in in vitro and in situ conditions. Int. J. Food Microbiol..

[bib107] Hossain F., Follett P., Vu K.D., Salmieri S., Senoussi C., Lacroix M. (2014). Radiosensitization of *Aspergillus niger* and *Penicillium chrysogenum* using basil essential oil and ionizing radiation for food decontamination. Food Control.

[bib108] Hu F., Tu X.F., Thakur K., Hu F., Li X.L., Zhang Y.S., Zhang J.G., Wei Z.J. (2019). Comparison of antifungal activity of essential oils from different plants against three fungi. Food Chem. Toxicol..

[bib109] Huang C., Qian Y., Viana T., Siegumfeldt H., Arneborg N., Larsen N., Jespersen L. (2020). The quorum-sensing molecule 2-phenylethanol impaired conidial germination, hyphal membrane integrity and growth of *Penicillium expansum* and *Penicillium nordicum*. J. Appl. Microbiol..

[bib110] Hyldgaard M., Mygind T., Meyer R.L. (2012). Essential oils in food preservation: mode of action, synergies, and interactions with food matrix components. Front. Microbiol..

[bib111] Hyun J.E., Bae Y.M., Yoon J.H., Lee S.Y. (2015). Preservative effectiveness of essential oils in vapor phase combined with modified atmosphere packaging against spoilage bacteria on fresh cabbage. Food Control.

[bib112] Ibusquiza P.S., Herrera J.J.R., Cabo M.L. (2011). Resistance to benzalkonium chloride, peracetic acid and nisin during formation of mature biofilms by *Listeria monocytogenes*. Food Microbiol..

[bib113] Jadhav S., Shah R., Bhave M., Palombo E.A. (2013). Inhibitory activity of yarrow essential oil on Listeria planktonic cells and biofilms. Food Control.

[bib114] Jamil B., Abbasi R., Abbasi S., Imran M., Khan S.U., Ihsan A., Javed S., Bokhari H., Imran M. (2016). Encapsulation of cardamom essential oil in chitosan nano-composites: in-vitro efficacy on antibiotic-resistant bacterial pathogens and cytotoxicity studies. Front. Microbiol..

[bib115] Jiang H., Zhong S., Schwarz P., Chen B., Rao J. (2022). Antifungal activity, mycotoxin inhibitory efficacy, and mode of action of hop essential oil nanoemulsion against Fusarium graminearum. Food Chem..

[bib116] Jiang X., Yu T., Liang Y., Ji S., Guo X., Ma J., Zhou L. (2016). Efflux pump-mediated benzalkonium chloride resistance in *Listeria monocytogenes* isolated from retail food. Int. J. Food Microbiol..

[bib117] Jiang Y.T., Yan P.F., Liang J.P. (2015). Biological changes of Enterococcus faecalis in the viable but nonculturable state. Genet. Mol. Res..

[bib118] Jiménez M., Domínguez J.A., Pascual-Pineda L.A., Azuara E., Beristain C.I. (2018). Elaboration and characterization of O/W cinnamon (Cinnamomum Zeylanicum) and black pepper (Piper Nigrum) emulsions. Food Hydrocolloids.

[bib119] Ju J., Chen X., Xie Y., Yu H., Guo Y., Cheng Y., Qian H., Yao W. (2019). Application of essential oil as a sustained release preparation in food packaging. Trends Food Sci. Technol..

[bib120] Kalagatur N.K., Mudili V., Kamasani J.R., Siddaiah C. (2018). Discrete and combined effects of ylang-ylang (cananga odorata) essential oil and gamma irradiation on growth and mycotoxins production by *Fusarium graminearum* in maize. Food Control.

[bib121] Kalemba D., Kunicka A. (2003). Antibacterial and antifungal properties of essential oils. Curr. Med. Chem..

[bib122] Karam L., Chehab R., Osaili T.M., Savvaidis I.N. (2020). Antimicrobial effect of thymol and carvacrol added to a vinegar-based marinade for controlling spoilage of marinated beef (shawarma) stored in air or vacuum packaging. Int. J. Food Microbiol..

[bib123] Karam L., Roustom R., Abiad M.G., El-Obeid T., Savvaidis I.N. (2019). Combined effects of thymol, carvacrol and packaging on the shelf-life of marinated chicken. Int. J. Food Microbiol..

[bib124] Karpanen T.J., Worthington T., Hendry E.R., Conway B.R., Lambert P.A. (2008). Antimicrobial efficacy of chlorhexidine digluconate alone and in combination with Eucalyptus oil, tea tree oil and thymol against planktonic and biofilm cultures of *Staphylococcus epidermidis*. J. Antimicrob. Chemother..

[bib125] Kavanaugh N.L., Ribbeck K. (2012). Selected antimicrobial essential oils eradicate *Pseudomonas* spp. and *Staphylococcus aureus* biofilms. Appl. Environ. Microbiol..

[bib126] Khan I., Bahuguna A., Kumar P., Bajpai V.K., Kang S.C. (2017). Antimicrobial potential of carvacrol against uropathogenic *Escherichia coli* via membrane disruption, depolarization, and reactive oxygen species generation. Front. Microbiol..

[bib127] Khelissa S.O., Abdallah M., Jama C., Faille C., Chihib N.E. (2017). Bacterial contamination and biofilm formation on abiotic surfaces and strategies to overcome their persistence. J. Mater. Environ. Sci..

[bib128] Khelissa S.O., Abdallah M., Jama C., Gharsallaoui A., Chihib N.E. (2019). Comparative study of growth temperature impact on the susceptibility of biofilm-detached and planktonic *Staphylococcus aureus* cells to benzalkonium chloride. Ann. Microbiol..

[bib129] Kibbee R.J., Ormeci B. (2017). Development of a sensitive and false-positive free PMA-qPCR viability assay to quantify VBNC *Escherichia coli* and evaluate disinfection performance in wastewater effluent. J. Microbiol. Methods.

[bib130] Kumar A., Kujur A., Yadav A., Pratap S., Prakash B. (2019). Optimization and mechanistic investigations on antifungal and Aflatoxin B1 inhibitory potential of nanoencapsulated plant-based bioactive compounds. Ind. Crop. Prod..

[bib131] Kwieciński J., Eick S., Wójcik K. (2009). Effects of tea tree (melaleuca alternifolia) oil on *Staphylococcus aureus* in biofilms and stationary growth phase. Int. J. Antimicrob. Agents.

[bib132] Kwon J.A., Yu C.B., Park H.D. (2003). Bacteriocidal effects and inhibition of cell separation of cinnamic aldehyde on *Bacillus cereus*. Lett. Appl. Microbiol..

[bib133] Lambert R.J.W., Skandamis P.N., Coote P.J., Nychas G.J.E. (2001). A study of the minimum inhibitory concentration and mode of action of oregano essential oil, thymol and carvacrol. J. Appl. Microbiol..

[bib134] Lavorgna M., Russo C., D’Abrosca B., Parrella A., Isidori M. (2016). Toxicity and genotoxicity of the quaternary ammonium compound benzalkonium chloride (BAC) using Daphnia magna and Ceriodaphnia dubia as model systems. Environ. Pollut..

[bib135] Ledwoch K., Dancer S.J., Otter J.A., Kerr K., Roposte D., Rushton L., Weiser R., Mahenthiralingam E., Muir D.D., Maillard J.Y. (2018). Beware biofilm! Dry biofilms containing bacterial pathogens on multiple healthcare surfaces; a multi-centre study. J. Hosp. Infect..

[bib136] Li Y.H., Tian X. (2012). Quorum sensing and bacterial social interactions in biofilms. Sensors.

[bib137] Liu J., Deng Y., Soteyome T., Li Y., Su J., Li L., Li B., Shirtliff M., Xu Z., Peters B. (2018). Induction and recovery of the viable but nonculturable state of hop-resistance Lactobacillus brevis. Front. Microbiol..

[bib138] Liu S., Tao M., Huang K. (2021). Encapsulation of Mānuka essential oil in yeast microcarriers for enhanced thermal stability and antimicrobial activity. Food Bioprocess Technol..

[bib139] López-Meneses A.K., Plascencia-Jatomea M., Lizardi-Mendoza J., Fernández-Quiroz D., Rodríguez-Félix F., Mourińo-Pérez R.R., Cortez-Rocha M.O. (2018). Schinus molle L. Essential oil-loaded chitosan nanoparticles: preparation, characterization, antifungal and anti-aflatoxigenic properties. LWT.

[bib140] Luciardi M.C., Blázquez M.A., Cartagena E., Bardón A., Arena M.E. (2016). Mandarin essential oils inhibit quorum sensing and virulence factors of *Pseudomonas aeruginosa*. LWT- Food Sci. Technol..

[bib141] Magnoli A.P., Poloni V.L., Cavaglieri L. (2019). Impact of mycotoxin contamination in the animal feed industry. Curr. Opin. Food Sci..

[bib142] Majeed M., Majeed S., Nagabhushanam K., Punnapuzha A., Philip S., Mundkur L. (2018). Rapid assessment of viable but nonculturable *Bacillus coagulans* MTCC 5856 in commercial formulations using flow cytometry. PLoS One.

[bib143] Mallozzi M., Viswanathan V.K., Vedantam G. (2010). Spore-forming Bacilli and Clostridia in human disease. Future Microbiol..

[bib144] Marino M., Bersani C., Comi G. (2001). Impedance measurements to study the antimicrobial activity of essential oils from lamiaceae and compositae. Int. J. Food Microbiol..

[bib145] Marotta S.M., Giarratana F., Parco A., Neri D., Ziino G., Giuffrida A., Panebianco A. (2016). Evaluation of the antibacterial activity of bergamo essential oils on different *Listeria monocytogenes* strains. Int. J. Food Saf..

[bib146] Maurya A., Prasad J., Das S., Dwivedy A.K. (2021). Essential oils and their application in food safety. Front. Sustain. Food Syst..

[bib147] Mechmechani S., Gharsallaoui A., Fadel A., Omari K.E., Khelissa S., Hamze M., Nour-Eddine N.E. (2022). Microencapsulation of carvacrol as an efficient tool to fight Pseudomonas aeruginosa and Enterococcus faecalis biofilms. PLoS One.

[bib148] Mechmechani S., Khelissa S., Gharsallaoui A., Omari K.E., Hamze M., Nour-Eddine N.E. (2022). Hurdle Technology using encapsulated enzymes and essential oils to fight bacterial biofilms. Appl. Microbiol. Biotechnol..

[bib149] Melin V.E., Melin T.E., Dessify B.J., Nguyen C.T., Shea C.S., Hrubec T.C. (2016). Quaternary ammonium disinfectants cause subfertility in mice by targeting both male and female reproductive processes. Reprod. Toxicol..

[bib150] Meng F.B., Gou Z.Z., Li Y.C., Zou L.H., Chen W.J., Liu D.Y. (2022). The efficiency of lemon essential oil-based nanoemulsions on the inhibition of Phomopsis sp. and reduction of postharvest decay of kiwifruit. Foods.

[bib151] Merino N., Berdejo D., Bento R., Salman H., Lanz M., Maggi F., Sánchez-Gómez S., García-Gonzalo D., Pagán R. (2019). Antimicrobial efficacy of thymbra capitata (L.) cav. Essential oil loaded in self-assembled Zein nanoparticles in combination with heat. Ind. Crop. Prod..

[bib152] Millan-Sango D., Garroni E., Farrugia C., Van Impe J.F.M., Valdramidis V.P. (2016). Determination of the efficacy of Ultrasound combined with essential oils on the decontamination of *Salmonella* inoculated lettuce leaves. LWT.

[bib153] Millezi A.F., das Graças Cardoso M., Alves E., Piccoli R.H. (2013). Reduction of *Aeromonas hidrophyla* biofilm on stainless stell surface by essential oils. Braz. J. Microbiol..

[bib154] Moghimi R., Aliahmadi A., Rafati H., Abtahi H.R., Amini S., Feizabadi M.M. (2018). Antibacterial and anti-biofilm activity of nanoemulsion of Thymus daenensis oil against multi-drug resistant *Acinetobacter baumannii*. J. Mol. Liq..

[bib155] Moghimi R., Ghaderi L., Rafati H., Aliahmadi A., McClements D.J. (2016). Superior antibacterial activity of nanoemulsion of Thymus daenensis essential oil against *E. coli*. Food Chem..

[bib156] Moosavy M.H., Basti A.A., Misaghi A., Salehi T.Z., Abbasifar R., Mousavi H.A.E., Alipour M., Razavi N.E., Gandomi H., Noori N. (2008). Effect of Zataria multiflora boiss. Essential oil and nisin on *Salmonella* typhimurium and *Staphylococcus aureus* in a food model system and on the bacterial cell membranes. Food Res. Int..

[bib157] Møretrø T., Schirmer B.C.T., Heir E., Fagerlund A., Hjemli P., Langsrud S. (2017). Tolerance to quaternary ammonium compound disinfectants may enhance growth of *Listeria monocytogenes* in the food industry. Int. J. Food Microbiol..

[bib158] Mortazavi N., Aliakbarlu J. (2019). Antibacterial effects of Ultrasound, cinnamon essential oil, and their combination against *Listeria monocytogenes* and *Salmonella* typhimurium in milk. J. Food Sci..

[bib159] Mukamolova G.V., Yanopolskaya N.D., Kell D.B., Kaprelyants A.S. (2018). On resuscitation from the dormant state of *Micrococcus luteus*. Antonie Leeuwenhoek.

[bib160] Mulder I., Siemens J., Sentek V., Amelung W., Smalla K., Jechalke S. (2018). Quaternary ammonium compounds in soil: implications for antibiotic resistance development. Rev. Environ. Sci. Biotechnol..

[bib161] Nahr F.K., Ghanbarzadeh B., Hamishehkar H., Kafil H.S. (2018). Food grade nanostructured lipid carrier for cardamom essential oil: preparation, characterization and antimicrobial activity. J. Funct.Foods.

[bib162] Narla A.V., Borenstein D.B., Wingreen N.S. (2021). A biophysical limit for quorum sensing in biofilms. Proc. Natl. Acad. Sci. U. S. A.

[bib163] Nogueira J.O., Campolina G.A., Batista L.R., Alves E., Caetano A.R.S., Brandão R.M., Nelson D.L., Cardoso M.D.G. (2021). Mechanism of action of various terpenes and phenylpropanoids against *Escherichia coli* and *Staphylococcus aureus*. FEMS Microbiol. Lett..

[bib164] Nostro A., Cellini L., Zimbalatti V., Blanco A.R., Marino A., Pizzimenti F., Di Giulio M., Bisignano G. (2012). Enhanced activity of carvacrol against biofilm of *Staphylococcus aureus* and *Staphylococcus epidermidis* in an acidic environment. APMIS.

[bib165] Nostro A., Procopio F., Pizzimenti F.C., Cannatelli M.A., Bisignano G., Marino A., Blanco A.R., Cioni P.L., Roccaro A.S. (2007). Effects of oregano, carvacrol and thymol on *Staphylococcus aureus* and *Staphylococcus epidermidis* biofilms. J. Med. Microbiol..

[bib166] Nuryastuti T., van der Mei H.C., Busscher H.J., Iravati S., Aman A.T., Krom B.P. (2009). Effect of cinnamon oil on IcaA expression and biofilm formation by *Staphylococcus epidermidis*. Appl. Environ. Microbiol..

[bib167] Omonijo F.A., Ni L., Gong J., Wang Q., Lahaye L., Yang C. (2018). Essential oils as alternatives to antibiotics in swine production. Anim. Nutr..

[bib168] Orlo E., Russo C., Nugnes R., Lavorgna M., Isidori M. (2021). Natural methoxyphenol compounds: antimicrobial activity against foodborne pathogens and food spoilage bacteria, and role in antioxidant processes. Food.

[bib169] Orta M.T., Noguez I., Casasola Rodriguez B., Roman Roman P.I. (2017). Effects of ozone and chlorine disinfection on VBNC Helicobacter pylori by molecular techniques and FESEM images. Environ. Technol..

[bib170] Palla M., Battini F., Cristani C., Giovannetti M., Squartini A., Agnolucci M. (2018). Quorum sensing in rhizobia isolated from the spores of the mycorrhizal symbiont rhizophagus intraradices. Mycorrhiza.

[bib171] Pathania R., Khan H., Kaushik R., Khan M.A. (2018). Essential oil nanoemulsions and their antimicrobial and food applications. Curr. Res. Nutr. Food Sci. J..

[bib172] Pérez-Conesa D., Cao J., Chen L., Mclandsborough L., Weiss J. (2011). Inactivation of *Listeria monocytogenes* and *Escherichia coli* O157:H7 biofilms by micelle-encapsulated eugenol and carvacrol. J. Food Protect..

[bib173] Pina-Pérez M.C., Martínez-López A., Rodrigo D. (2012). Cinnamon antimicrobial effect against *Salmonella* typhimurium cells treated by pulsed electric fields (PEF) in pasteurized skim milk beverage. Food Res. Int..

[bib174] Preda V.G., Sãndulescu O. (2019). Communication is the key: biofilms, quorum sensing, formation and prevention. Discoveries.

[bib175] Popiolski T.M., Otsuka I., Halila S., Muniz E.C., Soldi V., Borsali R. (2016). Preparation of polymeric micelles of poly(ethylene oxide-b-lactic acid) and their encapsulation with lavender oil. Mater. Res..

[bib176] Potts M. (2001). Desiccation tolerance: a simple process?. Trends Microbiol..

[bib177] Prakash A., Baskaran R., Paramasivam N., Vadivel V. (2018). Essential oil based nanoemulsions to improve the quality of minimally processed fruits and vegetables: Q review. Food Res. Int..

[bib178] Quendera A.P., Barreto A.S., Semedo-Lemsaddek T. (2019). Antimicrobial activity of essential oils against foodborne multidrug-resistant enterococci and aeromonads in planktonic and biofilm state. Food Sci. Technol. Int..

[bib179] Radünz M., Helbig E., Borges C.D., Gandra T.V., Gandra E.A. (2018). A mini-review on encapsulation of essential oils. J. Anal. Pharm. Res..

[bib180] Rajkowska K., Nowak A., Kunicka-Styczyńska A., Siadura A. (2016). Biological effects of various chemically characterized essential oils: investigation of the mode of action of *Candida albicans* and HeLa cells. RSC Adv..

[bib181] Ramirez M.L., Cendoya E., Nichea M.J., Zachetti V.G.L., Chulze S.N. (2018). Impact of toxigenic fungi and mycotoxins in chickpea: a review. Curr. Opin. Food Sci..

[bib182] Rattanachaikunsopon P., Phumkhachorn P. (2010). Assessment of factors influencing antimicrobial activity of carvacrol and cymene against *Vibrio cholerae* in food. J. Biosci. Bioeng..

[bib183] Rosato A., Sblano S., Salvagno L., Carocci A., Clodovea M.L., Corbo F., Fracchiolla G. (2020). Anti-biofilm inhibitory synergistic effects of combinations of essential oils and antibiotics. Antibiotics.

[bib184] Rossi G.G., Guterres K.B., Bonez P.C., da Silva Gundel S., Aggertt V.A., Siqueira F.S., Ourique A.F., Wagnerd R., Klein B., Santos R.C.V., de Campos M.M.A. (2017). Antibiofilm activity of nanoemulsions of cymbopogon flexuosus against rapidly growing Mycobacteria. Microb. Pathog..

[bib185] Russo C., Kundi M., Lavorgna M., Parrella A., Isidori M. (2018). Benzalkonium chloride and anticancer drugs in binary mixtures: reproductive toxicity and genotoxicity in the freshwater Crustacean Ceriodaphnia dubia. Arch. Environ. Contam. Toxicol..

[bib186] Sadekuzzaman M., Mizan M.F.R., Kim H.S., Yang S., Ha S.D. (2018). Activity of thyme and tea tree essential oils against selected foodborne pathogens in biofilms on abiotic surfaces. LWT.

[bib187] Sales A.J., Bagherizadeh Y., Malekzadeh P., Ahmadi B., Bonab F.R. (2017). Evaluation of the antimicrobial effects of essential oil of reseda lutea L. On pathogenic bacteria: *Staphylococcus aureus, Staphylococcus epidermidis*, and *Escherichia coli*. Arch. Clin. Microbiol..

[bib188] Sandasi M., Leonard C.M., Van Vuuren S.F., Viljoen A.M. (2011). Peppermint (mentha piperita) inhibits microbial biofilms in vitro. South Afr. J. Bot..

[bib189] Scharff R.L. (2012). Economic burden from health losses due to foodborne illness in the United States. J. Food Protect..

[bib190] Scharff R.L. (2018).

[bib191] Schottroff F., Frohling A., Zunabovic-Pichler M., Krottenthaler A., Schluter O., Jager H. (2018). Sublethal injury and viable but non-culturable (VBNC) state in microorganisms during preservation of food and biological materials by non-thermal processes. Front. Microbiol..

[bib193] Scotti R., Stringaro A., Nicolini L., Zanellato M., Boccia P., Maggi F., Gabbianelli R. (2021). Effects of essential oils from cymbopogon spp. and cinnamomum verum on biofilm and virulence properties of *Escherichia coli* O157:H7. Antibiotics.

[bib194] Setlow P. (2014). Germination of spores of Bacillus species: what we know and do not know. J. Bacteriol..

[bib195] Shetta A., Kegere J., Mamdouh W. (2019). Comparative study of encapsulated peppermint and green tea essential oils in chitosan nanoparticles: encapsulation, thermal stability, in-vitro release, antioxidant and antibacterial activities. Int. J. Biol. Macromol..

[bib196] Shi C., Jia Z., Sun Y., Chen Y., Guo D., Liu Z., Wen Q., Guo X., Ma L., Yang B., Baloch A.B., Xia X. (2017). Inactivation of nondesiccated and desiccated *Cronobacter sakazakii* in reconstituted infant formula by combination of citral and mild heat. J. Food Protect..

[bib198] Sieniawska E., Los R., Baj T., Malm A., Glowniak K. (2013). Antimicrobial efficacy of mutellina purpurea essential oil and α-pinene against *Staphylococcus epidermidis* grown in planktonic and biofilm cultures. Ind. Crop. Prod..

[bib199] Silva-Angulo A.B., Zanini S.F., Rosenthal A., Rodrigo D., Klein G., Martínez A. (2015). Comparative study of the effects of citral on the growth and injury of *Listeria innocua* and *Listeria monocytogenes* cells. PLoS One.

[bib200] Silva-Espinoza B.A., Palomares-Navarro J.J., Tapia-Rodriguez M.R., Cruz-Valenzuela M.R., González-Aguilar G.A., Silva-Campa E., Pedroza-Montero M., Almeida-Lopes M., Miranda R., Ayala-Zavala J.F. (2020). Combination of ultraviolet light-C and clove essential oil to inactivate *Salmonella* typhimurium biofilms on stainless steel. J. Food Saf..

[bib201] Singh A., Singh R.K., Bhunia A.K., Singh N. (2003). Efficacy of plant essential oils as antimicrobial agents against *Listeria monocytogenes* in hotdogs. LWT - Food Sci. Technol..

[bib202] Singh B.K., Tiwari S., Dubey N.K. (2021). Essential oils and their nanoformulations as green preservatives to boost food safety against mycotoxin contamination of food commodities: a review. J. Sci. Food Agric..

[bib203] Skandamis P., Tsigarida E., E Nychas G.J. (2002). The effect of oregano essential oil on survival/death of *Salmonella* typhimurium in meat stored at 5°C under aerobic, VP/MAP conditions. Food Microbiol..

[bib204] Soliman E.A., El-Moghazy A.Y., Mohy El-Din M.S., Massoud M.A. (2013). Microencapsulation of essential oils within alginate: formulation and in vitro evaluation of antifungal activity. J. Encapsulation Adsorpt. Sci..

[bib205] Somrani M., Debbabi H., Palop A. (2021). Antibacterial and antibiofilm activity of essential oil of clove against *Listeria monocytogenes* and *Salmonella enteritidis*. Food Sci. Technol. Int..

[bib206] Soni K.A., Oladunjoye A., Nannapaneni R., Schilling M.W., Silva J.L., Mikel B., Bailey R.H. (2013). Inhibition and inactivation of *Salmonella* typhimurium biofilms from polystyrene and stainless steel surfaces by essential oils and phenolic constituent carvacrol. J. Food Protect..

[bib207] Sreevidya V.S., Lenz K.A., Svoboda K.R., Ma H. (2018). Benzalkonium chloride, benzethonium chloride, and chloroxylenol - three replacement antimicrobials are more toxic than triclosan and triclocarban in two model organisms. Environ. Pollut..

[bib208] Srey S., Jahid I.K., Ha S.D. (2013). Biofilm Formation in food industries: a food safety concern. Food Control.

[bib209] Swamy M.K., Akhtar M.S., Sinniah U.R. (2016). Antimicrobial properties of plant essential oils against human pathogens and their mode of action: an updated review. Evid. base Compl. Alternative Med..

[bib210] Tang C., Chen J., Zhang L., Zhang R., Zhang S., Ye S., Zhao Z., Yang D. (2020). Exploring the antibacterial mechanism of essential oils by membrane permeability, apoptosis and biofilm formation combination with proteomics analysis against mecithillin-resistant *Staphylococcus aureus*. Int. J. Med. Microbiol..

[bib211] Tariq S., Wani S., Rasool W., Shafi K., Bhat M.A., Prabhakar A. (2019). A comprehensive review of the antibacterial, antifungal and antiviral potential of essential oils and their chemical constituents against drug-resistant microbial pathogens. Microb. Pathog..

[bib212] Tawema P., Han J., Vu K.D., Salmieri S., Lacroix M. (2016). Antimicrobial effects of combined UV-C or gamma radiation with natural antimicrobial formulations against *Listeria monocytogenes, Escherichia coli* O157: H7, and total yeasts/molds in fresh cut cauliflower. LWT - Food Sci. Technol..

[bib213] Teixeira R.F., Filho C.A.B., Borges C.D. (2022). Essential oils as natural antimicrobials for application in edible coatings for minimally processed apple and melon: a review on antimicrobial activity and characteristics of food models. Food Packag. Shelf Life.

[bib214] Trindade L.A., Oliveira J.D.A., de Castro R.D., Lima E.D.O. (2015). Inhibition of adherence of *C. albicans* to dental implants and cover screws by cymbopogon nardus essential oil and citronellal. Clin. Oral Invest..

[bib215] Trunet C., Carlin F., Coroller L. (2017). Investigating germination and outgrowth of bacterial spores at several scales. Trends Food Sci. Technol..

[bib216] Tsigarida E., Nychas G.J.E. (2001). Ecophysiological attributes of a Lactobacillus sp. and a Pseudomonas sp. on sterile beef fillets in relation to storage temperature and film permeability. J. Appl. Microbiol..

[bib217] Turgis M., Han J., Caillet S., Lacroix M. (2009). Antimicrobial activity of mustard essential oil against *Escherichia coli* O157:H7 and *Salmonella typhi*. Food Control.

[bib218] Ultee A., Bennik M.H.J., Moezelaar R. (2002). The phenolic hydroxyl group of carvacrol is essential for action against the food-borne pathogen *Bacillus cereus*. Appl. Environ. Microbiol..

[bib219] Ultee A., Smid E.J. (2001). Influence of carvacrol on growth and toxin production by *Bacillus cereus*. Int. J. Food Microbiol..

[bib220] Valeriano C., de Oliveira T.L.C., de Carvalho S.M., Cardoso M.D.G., Alves E., Piccoli R.H. (2012). The sanitizing action of essential oil-based solutions against *Salmonella enterica* serotype enteritidis S64 biofilm formation on AISI 304 stainless steel. Food Control.

[bib221] Valková V., Dúranová H., Galovičová L., Vukovic N.L., Vukic M., Kačániová M. (2021). In vitro antimicrobial activity of lavender, mint, and rosemary essential oils and the effect of their vapours on growth of Penicillium spp. in a bread model system. Molecules.

[bib222] Vesty E.F., Whitbread E., Needs A.L., Tanko S., Jones W., Halliday K., Ghaderiardakani F., Xiaoguang L., Cámara M., Coates J. (2020). Cross-kingdom signalling regulates spore germination in the moss physcomitrella patens. Sci. Rep..

[bib223] Vetas D., Dimitropoulou E., Mitropoulou G., Kourkoutas Y., Giaouris E. (2017). Disinfection efficiencies of sage and spearmint essential oils against planktonic and biofilm *Staphylococcus aureus* cells in comparison with sodium hypochlorite. Int. J. Food Microbiol..

[bib224] Vidács A., Kerekes E., Rajkó R., Petkovits T., Alharbi N.S., Khaled J.M., Vágvölgyi C., Krisch J. (2018). Optimization of essential oil-based natural disinfectants against *Listeria monocytogenes* and *Escherichia coli* biofilms formed on polypropylene surfaces. J. Mol. Liq..

[bib225] Walsh S.E., Maillard J.Y., Russell A.D., Catrenich C.E., Charbonneau D.L., Bartolo R.G. (2003). Activity and mechanisms of action of selected biocidal agents on gram-positive and -negative bacteria. J. Appl. Microbiol..

[bib226] Wang L., Xia Q., Li Y. (2018). Label free-based proteomic analysis of proteins in *Bacillus cereus* spores regulated by high pressure processing and slightly acidic electrolyzed water treatment. Food Control.

[bib228] Wells-Bennik M.H.J., Eijlander R.T., den Besten H.M.W., Berendsen E.M., Warda A.K., Krawczyk A.O., Nierop Groot M.N., Xiao Y., Zwietering M.H., Kuipers O.P., Abee T. (2016). Bacterial spores in food: survival, emergence, and outgrowth. Annu. Rev. Food Sci. Technol..

[bib229] Wu D., Lu J., Zhong S., Schwarz P., Chen B., Rao J. (2019). Influence of nonionic and ionic surfactants on the antifungal and mycotoxin inhibitory efficacy of cinnamon oil nanoemulsions. Food Funct..

[bib230] Wu H., Zhang H., Wang C., Wu Y., Xie J., Jin X., Yang J., Ye J. (2011). Genoprotective effect of hyaluronic acid against benzalkonium chloride-induced DNA damage in human corneal epithelial cells. Mol. Vis..

[bib231] Xiong Q., Zhang H., Shu X., Sun X., Feng H., Xu Z., Kovács A., Yunpeng L., Zhang R. (2021). Quorum sensing signal autoinducer-2 inhibits sporulation of Bacillus by interacting with RapC and functions across species. bioRxiv.

[bib232] Xu D., Wei M., Peng S., Mo H., Huang L., Yao L., Hu L. (2021). Cuminaldehyde in cumin essential oils prevents the growth and Aflatoxin B1 biosynthesis of Aspergillus flavus in peanuts. Food Control.

[bib233] Xu J., Zhou F., Ji B.P., Pei R.S., Xu N. (2008). The antibacterial mechanism of carvacrol and thymol against *Escherichia coli*. Lett. Appl. Microbiol..

[bib234] Yamazaki K., Yamamoto T., Kawai Y., Inoue N. (2004). Enhancement of antilisterial activity of essential oil constituents by nisin and diglycerol fatty acid ester. Food Microbiol..

[bib235] Yammine J., Gharsallaoui A., Fadel A., Mechmechani S., Karam L., Ismail A., E Chihib N. (2022). Enhanced antimicrobial, antibiofilm and ecotoxic activities of nanoencapsulated carvacrol and thymol as compared to their free counterparts. Food Control.

[bib236] Yoon J.I., Bajpai V.K., Kang S.C. (2011). Synergistic effect of nisin and cone essential oil of metasequoia glyptostroboides miki ex Hu against *Listeria monocytogenes* in milk samples. Food Chem. Toxicol..

[bib237] Zhang C., Cui F., Zeng G.M., Jiang M., Yang Z.Z., Yu Z.G., Zhu M.Y., Shen L.Q. (2015). Quaternary ammonium compounds (QACs): a review on occurrence, fate and toxicity in the environment. Sci. Total Environ..

[bib238] Zhang J., Ye K.P., Zhang X., Pan D.D., Sun Y.Y., Cao J.X. (2017). Antibacterial activity and mechanism of action of black pepper essential oil on meat-borne *Escherichia coli*. Front. Microbiol..

[bib240] Zhao F., Wang Y., An H., Hao Y., Hu X., Liao X. (2016). New insights into the formation of viable but nonculturable *Escherichia coli* O157:H7 induced by high-pressure CO_2_. mBio.

[bib241] Zhong J., Zhao X. (2018). Detection of viable but non-culturable *Escherichia coli* O157:H7 by PCR in combination with propidium monoazide. Biotech.

